# Remanufacturing and channel strategies in e-commerce closed-loop supply chain

**DOI:** 10.1371/journal.pone.0303447

**Published:** 2024-05-16

**Authors:** Ying Shi, Rong Ma, Tianjian Yang

**Affiliations:** 1 China Telecom Research Institute, Guangzhou, 510630, China; 2 School of Economics and Management, Beijing University of Posts and Telecommunications, Beijing, 100876, China; 3 School of Modern Post (School of Automation), Beijing University of Posts and Telecommunications, Beijing, 100876, China; Management and Science University Faculty of Business Management and Professional Studies, MALAYSIA

## Abstract

This paper studies the recycling and remanufacturing mode and sales channel issues in the closed-loop supply chain. Specifically, this study establishes an e-commerce closed-loop supply chain consisting of a manufacturer and an e-commerce platform, and divides the recycling model into recycling by the manufacturer or recycling by the platform. Considering two common sales models in e-commerce platforms: the resale model and agency model, combined with the recycling model, four different research scenarios are formed. We use backward induction to solve the Stackelberg game problem and explore the remanufacturing and channel strategies of the manufacturer and the e-commerce platform. The research results show that for the manufacturer, under the same recycling model, when consumers’ preference for remanufactured products and the sensitivity of recycling volume to recycling prices are low, he will prefer the resale model. Under the same sales model, the manufacturer always prefers the recycling model in which he is responsible for recycling. However, the choice of platform is contrary to that of the manufacturer. In the resale model, both the manufacturer and the platform will choose to recycle by themselves, which cannot achieve a win-win situation. Under the agency model, when consumers’ preference for remanufactured products is high and the sensitivity coefficient of recycling volume to recycling price is low, supply chain members can achieve a win-win situation, and the scope of the win-win situation decreases as the unit production cost of new products increases. In addition, rising consumer preference for remanufactured products will lead to lower consumer surplus.

## 1. Introduction

Research on supply chains has long had a strong foundation in sustainable development, and many studies have been conducted based on closed-loop supply chains. A closed-loop supply chain (CLSC) refers to the recycling of goods from consumers and the reuse of all or part of the goods to obtain added value. Guide and Van Wassenhove define a closed-loop supply chain from a business perspective as: a system designed, controlled and operated to maximize value creation throughout a product’s lifecycle and to dynamically recover value from different types and quantities of returns over time [1]. Recently, the "China Renewable Resource Recycling Industry Development Report (2023)" released by the China National Resources Recycling Association pointed out that the total volume of renewable resource recycling in China in 2022 was approximately 371 million tons [2]. This number highlights the importance of renewable resource recycling and its huge potential for the sustainable development of our country.

The continuous development of the Internet has promoted the development of the e-commerce market. According to the "China E-Commerce Report (2022)" released by the Ministry of Commerce, data from the National Bureau of Statistics of China show that in 2022, the national e-commerce transaction volume reached 43.83 trillion yuan, which is an increase of 3.5% over the previous year. The discussion on CLSC has expanded to include the context of e-commerce due to its rapid growth. Product recycling and the sale of remanufactured products are common in e-commerce, and there are different recycling entities. On the one hand, e-platforms can be responsible for recycling. For example, large e-commerce platforms JD.com and Taobao both provide recycling services to consumers, including door-to-door pickup and in-person quality inspection services. There are also specialized second-hand product recycling platforms, such as Xianyu and Zhuanzhuan. On the other hand, as the main body of product manufacturing and sales, manufacturers may also serve for recycling. For example, Bright Milk recycles milk cartons, and Uniqlo also announced product recycling and reuse activities on its official website. Moreover, to encourage consumers to recycle products, some manufacturers have also developed recycling incentive mechanisms. For example, the German water purifier brand BRITA recycles filter elements from consumers. Scores can be accumulated based on the number of recycling filters, which can be used to redeem new water purifier filter elements. It can be seen that different recycling models are both common in CLSC. Trade-in services are also a common recycling incentive mechanism, where consumers can exchange old (used) products for new ones [3].

In the e-commerce platform supply chain, in addition to the selection of recycling and remanufacturing entities, there is also an important business strategy, that is, the selection of sales modes. Existing studies have discussed the CLSC decision-making issues of different recycling entities, but did not consider the different sales modes in e-commerce platforms [4]. The resale mode and agency sales mode are the two main sales models on e-commerce platforms. As an online retailer, the platform purchases goods wholesale from manufacturers and resells them to customers. This is a resale mode, such as JD.com’s self-operated sales. The e-commerce platform operates as an online market, offering customers and manufacturers a trading platform and charging manufacturers commissions. This is an agency mode, such as the Taobao platform. In the agency mode, producers can interact directly with customers, whereas in the resale mode, the only role is to produce goods and wholesale them to the platform. What impact will different distances from consumers have on the choice of recycling mode? Is there a relationship between the way manufacturers sell their products and their choice of recycling mode? There is no clear conclusion yet on this issue. China’s two leading e-commerce platforms, JD.com and Taobao platforms both provide product recycling services to consumers. There is a lack of discussion on how different recycling models perform under different sales modes, and what impact different model combinations have on the profitability of supply chain members.

This study seeks to answer the following questions in light of the aforementioned theoretical and practical motivations:

(1) In the e-commerce closed-loop supply chain, what are the optimal recycling models for the manufacturer and the e-commerce platform under various sales models?

(2) Faced with the same recycling model, how do supply chain participants make channel strategies?

(3) Is there a strategy acceptable to both supply chain members to create a win-win scenario?

(4) What impact will different strategies have on consumer surplus?

To solve these problems, this paper establishes an e-commerce closed-loop supply chain model consisting of an e-commerce platform and a manufacturer. Considering two recycling models, the manufacturer is responsible for recycling and the e-commerce platform is responsible for recycling, as well as the two sales models of resale and agency on the e-commerce platform. Combining different strategies, four research scenarios are constructed. We establish the profit functions of the manufacturer and the e-commerce platform in each scenario respectively, and obtained the equilibrium solutions in each scenario by solving the Stackelberg game problem. By comparing the equilibrium solutions of different scenarios, the strategic choices of supply chain members are obtained. This study discusses the different sales models of the e-commerce platform from the perspective of different recycling entities in the closed-loop supply chain, supplementing the shortcomings of the existing literature by offering management insights into the operational choices made by participants in the closed-loop supply chain for e-commerce.

The remainder of this paper includes the following. Section 2 summarizes the literature relevant to this study. Section 3 explains the establishment of the model. The four models are discussed in detail in Section 4. Section 5 discusses the strategies of supply chain members. Section 6 compares the consumer surpluses in four models. The study findings are summarized in Section 7.

## 2. Literature review

The literature related to this study is mainly about the closed-loop supply chain and e-commerce sales modes.

### 2.1. Closed-loop supply chain

Savaskan et al. studied the closed-loop supply chain problem considering product remanufacturing, considered three different recycling modes and designed a coordination mechanism [5]. Many studies on CLSC are based on this. The closed-loop supply chain was defined by Guide and Van Wassenhove [1] and how it was developing from a commercial standpoint was summed up.

Scholars have discussed CLSC issues in a competitive environment. When there are competing retailers in the supply chain, supply chain members have different choices for direct and indirect recycling modes in decentralized and closed-loop supply chain structures [6]. Manufacturers have an incentive to remanufacture when there are competitors in the supply chain, but when competition declines, this incentive will diminish. In contrast, service competition hurts both competing manufacturers, while price competition might boost remanufacturers’ profitability [7]. A three-level CLSC network model with competition was developed by Qiang et al., and the effects of competition intensity and other variables on equilibrium were explored [8]. Both direct horizontal competition between two manufacturers and vertical competition between manufacturers and retailers were examined by Patare and Venkataraman [9]. They discovered that a higher level of competition can raise product quality.

As an important part of CLSC, consumer behavior has also attracted the attention of scholars. To optimize their utility, consumers may have varied preferences for different products due to the differences between new and remanufactured products. Scholars have done some studies taking into account customer preferences. The ideal price for both new and remanufactured goods was investigated by Abbey et al. using data from large-scale trials conducted in consumer preference models [10]. A rise in customer demand for remanufactured goods can boost supply chain efficiency overall and positively affect both product and price points. However, remanufactured products will suffer if manufacturers penalize e-commerce platforms given that they are concerned about fairness [11]. The proportion of green consumers among total consumers and their green preferences are also beneficial to manufacturers’ economic benefits and have an important impact on manufacturers’ green product segmentation strategies [12]. Due to the existence of consumer preferences, the factors that affect consumers’ willingness to purchase remanufactured products are also a question worth studying. Agrawal et al. conducted a behavioral experiment [13]. Hazen et al. examined customers’ propensity to switch from buying new items to buying remanufactured ones by combining macro-level pricing, government incentives, and environmental benefit variables with the moderating effects of micro-level consumer attitudes [14]. Consumers’ propensity to buy remanufactured products is greatly influenced by several factors, including perceived risks, costs, and moral obligations and responsibilities [15].

Due to the supervision of corporate social responsibility and the pursuit of sustainable social and economic development, the government also plays an important role in CLSC, which has attracted the attention of scholars [16,17]. The government’s management of CLSC mainly has two aspects, one is supervision and the other is subsidies. In terms of government regulation, the influence of recycling regulations on the remanufacturing business was examined by Esenduran et al. [18]. The trading supervision of carbon emission reductions by the government influences the decisions made in CLSC. By controlling the price of carbon trading, the government can have an impact on supply chain choices [19]. Carbon taxes are also a way for governments to regulate in order to promote sustainable development. Ma and Liu [20], and Li et al. [21] both discussed the carbon tax issue. Wang and Hong analyzed the best pricing and recycling practices in a CLSC with two recycling channels where the government provides subsidies to the manufacturer or two recyclers [22]. They found that subsidy policies stimulate consumption and increase the profits of supply chain members. Furthermore, government subsidies can regulate CLSC’s profit distribution. The effect of the subsidy will depend on the subsidy rate as well as the subsidy ceiling [23].

An essential component of CLSC is returns. Its objective is to recycle waste or products that don’t satisfy customer expectations from customers to a specific point in the supply chain where they can be processed. Researchers have studied return policies in great detail [24,25]. Additional shipping charges are frequently associated with the return process and are typically covered by the customer or retailer. It makes sense for customers to shoulder the cost when considering the real number of returns—that is, whether the volume of returns is high or the percentage of non-defective products among the returned products is low [26]. Zhang et al. conducted a study on the issue of defective products and waste returns. They concluded that, to lower the rate of defective product returns, it is not desirable to incur significant costs to improve product quality [27]. The best return policies vary depending on the supply contract. Comparative analysis revealed that buy-back and wholesale pricing contracts have stricter return policies than quantity discount contracts, which also result in higher demand and return requirements [28]. In an effort to encourage consumers to adopt sustainable practices, managers have created various programs for recycling used goods. To confirm the efficacy of these programs, Taleizadeh et al. set up various scenarios for examination. They discovered that acceptable return rules can accomplish supply chain coordination as well as environmental protection [29].

The various entities in charge of recycling and remanufacturing in CLSC have been covered in studies. Recycling activities can be carried out in a CLSC under cap-and-trade regulation by retailers, manufacturers, or other parties, and the carbon emissions under various regimes vary [30]. Manufacturers or sellers are able to remanufacture waste WEEE products under the WEEE CLSC. The best options for remanufacturing companies vary depending on the funding policies of the government [31].

Scholars have also addressed many aspects of the e-commerce CLSC, including platforms that offer extra warranty services, low-carbon e-commerce closed-loop supply chains, and differential pricing [11,32,33]. Blockchain technology is closely related to e-commerce. Ma and Hu talked about how platforms could maximize the synergy between "blockchain + sales formats" to enhance CLSC’s ESS (economic, social, and environmental) performance [34]. Liu et al. discussed the recycling channel structure selection problem of the e-commerce CLSC. The difference from this study is that they discussed three different recycling models, but did not consider different sales models [35].

Although studies related to CLSC have considered different recycling and manufacturing entities and have also studied channel issues, they haven’t looked at the topic of e-commerce-related sales channel strategies.

### 2.2. E-commerce sales mode

The resale model and agency model are the two main sales models in e-commerce. How to choose the optimal model is an important strategic issue faced by supply chain members. Abhishek et al. constructed a stylized theoretical framework to solve a crucial issue for e-tailers: When should an agency sales strategy be employed rather than a more traditional resale approach [36]? Based on this, many studies on e-commerce sales modes have been carried out. Tian et al. compared the performance of different sales modes in the e-commerce supply chain, offering guidance for mode selection and important references for subsequent research [37]. Diverse aspects have been incorporated by scholars into the channel models research. Manufacturers tend to select the agency model when there are detrimental effects from online channels to offline channels and the online channels are not competitive [38]. The choice of channels by manufacturers is significantly influenced by the presence of the secondary market. One important consideration is the price differential between new and old products. Manufacturers who want to sell expensive products will opt for the reselling model if there is a secondary market [39]. Hu et al. further studied the interaction between suppliers’ strategies for introducing market channels and e-tailers’ sales mode choices, they found that channel competition also affects the choice of sales model [40].

Related research has also extended to the situation with more supply chain members. In this case, there is generally horizontal competition between manufacturers or retailers. Researchers looked at the case of an e-commerce platform with several competitors and concluded that it makes more sense for manufacturers to choose resale on the platform rather than sell directly to customers. Simultaneously, if a manufacturer just opts for direct sales channels, then another manufacturer ought to run both platform resale channels and direct sales channels [41,42]. In contrast to platforms, Wang et al. talked about the emergence of independent internet retailers. The findings of the study indicate that when direct sales are low-cost, manufacturers will opt for them. Otherwise, commissions and competition have an impact on the choice of agency and resale models [43]. The above studies are all based on information symmetry, and scholars have also discussed scenarios of information asymmetry [44,45]. Sun et al. investigated the influence of sales form [46]. Under different sales structures, e-commerce platforms have varying incentives to reveal demand information to upstream. For instance, under the agency model, information regarding negative demand should not be shared, and information about strong market demand should not be disclosed during resale [47].

Hot issues such as logistics services and live sales in e-commerce are inseparable from discussions combined with sales models [48]. The platform’s choice of sales model will be influenced by its level of logistics. The hybrid sales model is the ideal option when the platform’s degree of logistics significantly surpasses that of third parties [49]. The platform’s decision to establish market channels is also affected by the level of logistics services. With high logistics service sales, platforms are more willing to introduce market channels [50]. In addition, as an emerging type of e-commerce, the issue of live streaming sales and e-commerce sales modes has also attracted the attention of scholars. Supply chain participants will select the agency model simultaneously when evaluating the competition and spillover impacts of live broadcasting under suitable commissions due to negative competition effects and low competition intensity [51].

Cao et al. studied the trade-in problem, considered the competition between the third-party seller and platform self-operation store, analyzed different trade-in models, and found the optimal trading strategy of the platform [52]. Yang et al. examined the issue of firms providing dual-channel trade-in programs and analyzed the impact of the profitability of dual-channel firms in the trade-in operation model [3].

Existing research lacks the issue of sales mode selection in e-commerce CLSC. The discrepancies between this research and relevant literature are presented in Table 1. As can be seen from the table, our research is close to that of Yang et al. [3], but they studied the issue of trade-in for old products and did not consider the sales of remanufactured products, nor did they discuss the combination of different sales models and recycling models. This study supplements the deficiencies of related research. We integrate the approaches of the channel model and the recycling model, considering the inclinations of consumers towards remanufactured goods. Based on the decision-making of supply chain members, win-win issues are discussed.

**Table 1:** A comparison of this research with relevant literature.

Authors	CLCS	E-platform supply chain	Sales mode		Responsible parties of recycling/remanufacturing	Consumer preference	CLSC members
			Resale mode	Agency mode			
Savaskan et al.[5]	✓				✓		M, R
Yang et al. [30]	✓				✓		M, R
Quan et al. [53]	✓				✓		M, R
Wang et al. [54]		✓				✓	M, R
Zhang et al. [31]	✓				✓	✓	M, R, TPR
Tian et al. [37]		✓	✓	✓			M, E
Wang et al. [32]	✓	✓		✓			M, E, G
Cui et al. [33]	✓	✓		✓			M, E
Qin et al. [55]	✓				✓	✓	M, TPR
Cao et al. [52]	✓	✓		✓		✓	R, E
Zhang and Ma [50]		✓	✓	✓		✓	M, E
Wang et al. [11]	✓	✓		✓		✓	M, E
Yang et al. [3]	✓	✓		✓	✓	✓	M, E, S
This study	✓	✓	✓	✓	✓	✓	M, E

Note: “M” represents manufacturer, “R” represents retailer, “TPR” represents third-party recycler, “E” represents e-commerce platform, “G” represents government, and “S” represents physical store.

## 3. Problem description and assumptions

**Fig 1:**
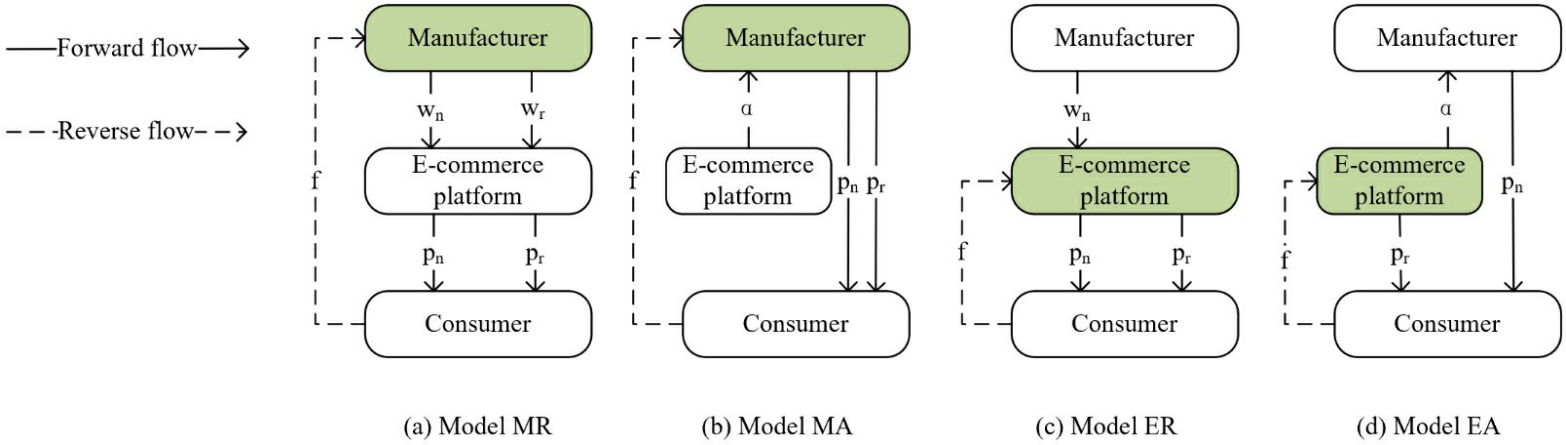
Different models.

As shown in Fig 1, We construct a closed-loop e-commerce supply chain that consists of an e-commerce platform (she) and a manufacturer (he). In the illustration, the forward product circulation direction is represented by the solid line, and the reverse product circulation direction is shown by the dotted line, and the green color represents the supply chain member responsible for recycling. The market offers both brand-new and remanufactured products. Based on different collection models and channel structures, four different scenarios are established: (a) Model MR: e-commerce platform resale model, the collection subject is the manufacturer. As shown in the figure, the manufacturer sells new and remanufactured products to the e-commerce platform at wholesale prices *w*_*n*_ and *w*_*r*_, and then the platform sells the products to consumers at resale prices *p*_*n*_ and *p*_*r*_. The manufacturer recycles the product to the consumer at price *f*. (b) Model MA: agency sales model, the collection subject is the manufacturer. The manufacturer sells the new and remanufactured products directly to consumers at resale prices *p*_*n*_ and *p*_*r*_, and the platform charges a certain percentage of commission *α*. The manufacturer recycles the product to the consumer at price *f*. (c) Model ER: e-commerce platform resale model, the collection subject is the e-commerce platform. The manufacturer sells the new product to the e-commerce platform at a wholesale price *w*_*n*_, and then the platform sells the new product to consumers at a resale price *p*_*n*_. The e-commerce platform recycles products to consumers at price *f* and sells remanufactured products directly to consumers at price *p*_*r*_. (d) Model EA: agency sales model, the collection subject is the e-commerce platform. The manufacturer sells the new products directly to consumers at resale prices *p*_*n*_, and the platform charges a certain percentage of commission *α*. The e-commerce platform recycles products to consumers at price *f* and sells remanufactured products directly to consumers at price *p*_*r*_.

### 3.1. Consumer demand

Referring to the research of Jia and Li and Wang et al., in the CLSC, it is assumed that the market size is 1, and consumers’ willingness to pay for a new product is *θ*, which is a uniform distribution between 0 and 1. This study considers ordinary consumers, not consumers with green preferences. Because they are unsure of the relative worth of remanufactured items over new ones, consumers have doubts about the quality [56]. So we assume that the buyer’s willingness to pay for a remanufactured product is a portion *β* of *θ* with *β*∈[0,1] [18,31]. The utility of a consumer buying a new product is *U*_*n*_ = *θ*−*p*_*n*_, and the utility of a consumer buying a remanufactured product is *U*_*r*_ = *βθ*−*p*_*r*_. Considering the principle of utility maximization, customers will buy a new product if *U*_*n*_≥*U*_*r*_ and *U*_*n*_≥0. This will result in a new product demand function $D_{n}=1-\frac{p_{n}-p_{r}}{1-\beta }$. If *U*_*r*_≥*U*_*n*_ and *U*_*r*_≥0, customers will buy a remanufactured product, creating a demand function for remanufactured products $D_{r}=\frac{p_{n}-p_{r}}{1-\beta }-\frac{p_{r}}{\beta }$ [4,57].

### 3.2. Production cost

The manufacturer’s unit costs of producing new and remanufactured goods are *c*_*n*_ and *c*_*r*_, respectively. Based on Zheng et al. we assume that *c*_*n*_>*c*_*r*_ = 0. That is, a new product’s unit cost is more than a remanufactured product’s [54,58].

### 3.3. Recycling cost

Referring to Gao et al., the product’s recycling quantity is *R* = *λf*, where *λ* is the product’s sensitivity coefficient to the recycling price and *f* is the unit recycling price. The recycling quantity is assumed to be equal to the demand for the remanufactured goods, i.e., *R* = *D*_*r*_. This gives = $\frac{D_{r}}{\lambda }$. So the cost of recycling is $\frac{1}{\lambda }{D_{r}}^{2}$ [59].

All notations and explanations are given in Table 2.

**Table 2:** Notations and explanations.

Notation	Explanation
*θ*	Consumers’ willingness to pay for a new product, *θ*∈[0,1]
*β*	Consumers’ willingness to pay for a remanufactured product, *β*∈[0,1]
*p*_*n*_	Price of the new product
*p*_*r*_	Price of the remanufactured product
*c*_*n*_	Unit production cost of a new product
*c*_*r*_	Unit production cost of a remanufactured product
*λ*	The recycling quantity’s sensitivity coefficient to the recycling price
*f*	Unit recycling price
*R*	The recycling quantity of the product
*U*_*n*_	Consumers’ Utility of buying a new product
*U*_*r*_	Consumers’ Utility of buying a remanufactured product
*D*_*n*_	Demand for the new product
*D*_*r*_	Demand for the remanufactured product
$\pi_{j}^{i}$	Profit of the manufacturer/e-commerce platform in different models, i = MR, MA, ER, EA, j = M, E.

## 4. Model and analyse

For the four different scenarios, we establish the profit function separately, and apply the Stackelberg game-theoretic model to obtain the equilibrium solutions by backward derivation and analyze the results.

### 4.1. Model MR

In Model MR, the manufacturer is responsible for recycling the products. The manufacturer produces new and remanufactured products and wholesales them to the e-commerce platform, which sells them to customers. Once a product’s life cycle is complete, the manufacturer gathers the items for remanufacturing.The decision sequence is: the manufacturer decides on the wholesale prices *w*_*n*_ and *w*_*r*_. The e-commerce platform then sets the resale prices *p*_*n*_ and *p*_*r*_. The profit functions for the manufacturer and the e-commerce platform are as follows:

$$\pi_{M}^{MR}=(w_{n}-c_{n})D_{n}+w_{r}D_{r}-\frac{1}{\lambda }{D_{r}}^{2}$$ (1)

$$\pi_{E}^{MR}=({p_{n}-w}_{n})D_{n}+({p_{r}-w}_{r})D_{r}$$ (2)

The following is the equilibrium solution obtained by backward induction:

$$w_{n}^{MR*}=\frac{1}{2}(1+c_{n}),w_{r}^{MR*}=\frac{\beta (1+2\beta \lambda -2\beta^{2}\lambda +c_{n})}{2+4\beta \lambda -4\beta^{2}\lambda },p_{n}^{MR*}=\frac{1}{4}(3+c_{n}),p_{r}^{MR*}=\frac{\beta (3+6\beta \lambda -6\beta^{2}\lambda +c_{n})}{4+8\beta \lambda -8\beta^{2}\lambda },$$

$$\pi_{M}^{MR*}=\frac{-1-2\beta \lambda +2\beta^{2}\lambda +(2+4\beta \lambda -4\beta^{2}\lambda )c_{n}-(1+2\beta \lambda )c_{n}^{2}}{8(-1-2\beta \lambda +2\beta^{2}\lambda )},$$

$$\pi_{E}^{MR*}=\frac{1}{16}(1-2c_{n}+\frac{(1+4\beta \lambda +4\beta^{2}(-1+\lambda )\lambda -4\beta^{3}\lambda^{2})c_{n}^{2}}{{\left(1+2\beta \lambda -2\beta^{2}\lambda \right)}^{2}})$$

See the appendix for proofs of all corollaries and propositions.

**Corollary 1.** (i). $\frac{\partial w_{n}^{MR*}}{\partial c_{n}}>0,\frac{\partial w_{r}^{MR*}}{\partial c_{n}}>0,\frac{\partial p_{n}^{MR*}}{\partial c_{n}}>0,\frac{\partial p_{r}^{MR*}}{\partial c_{n}}>0$;

(ii). $\frac{\partial w_{r}^{MR*}}{\partial \beta }>0,\frac{\partial p_{r}^{MR*}}{\partial \beta }>0$;

(iii). $\frac{\partial w_{r}^{MR*}}{\partial \lambda }<0,\frac{\partial p_{r}^{MR*}}{\partial \lambda }<0$.

Corollary 1 states that the wholesale price and sales price of the new product and the remanufactured product are positively correlated with the unit production cost of the new product. The impact of an increase in new product production costs on new product prices is in line with pricing strategies. To achieve profitability, the wholesale price set by the manufacturer will rise, and this will impact the platform retailer’s sales pricing. The increase in the price of the new product requires the manufacturer to adjust that of the remanufactured good at the same time to keep the competitive relationship between the two products in the market. The wholesale and sales prices of the remanufactured product are positively correlated with consumer preference for the remanufactured product, and negatively related to the sensitivity coefficient of recycling volume to recycling price. It makes sense for customer choices to have an influence on product prices. A higher value of *λ* results in a lower recycling price for the remanufactured product. This lowers the cost of the remanufacturing process for the producer and ultimately lowers the remanufactured product’s wholesale price. The remanufactured product’s sales price decreases as well.

### 4.2. Model MA

In model MA, the product’s recycling is the manufacturer’s responsibility. The manufacturer produces new and remanufactured products for direct sale to consumers. The manufacturer pays a commission to the e-commerce platform, which serves as an online marketplace. When a product reaches the end of its life cycle, the manufacturer gathers it for remanufacturing. The decision sequence is: the manufacturer decides the selling price *p*_*n*_ and *p*_*r*_. The profit functions for the supply chain members are as follows:

$$\pi_{M}^{MA}=({(1-\alpha )p}_{n}-c_{n})D_{n}+{(1-\alpha )p}_{r}D_{r}-\frac{1}{\lambda }{D_{r}}^{2}$$ (3)

$$\pi_{E}^{MA}={\alpha p}_{n}D_{n}+{\alpha p}_{r}D_{r}$$ (4)

The equilibrium price and profit are as follows:

$$p_{n}^{MA*}=\frac{1-\alpha +c_{n}}{2-2\alpha },p_{r}^{MA*}=\frac{1}{2}\beta \left(1-\frac{c_{n}}{\left(-1+\alpha )(1+(-1+\alpha )\beta^{2}\lambda +\beta (\lambda -\alpha \lambda )\right)}\right),$$

$$\pi_{M}^{MA*}=\frac{1}{4}\left(1-\alpha -2c_{n}+\frac{(-1+(-1+\alpha )\beta \lambda )c_{n}^{2}}{\left(-1+\alpha )(1+(-1+\alpha )\beta^{2}\lambda +\beta (\lambda -\alpha \lambda )\right)}\right),$$

$$\pi_{E}^{MA*}=\frac{1}{4}\alpha (1+\frac{(-1+2(-1+\alpha )\beta \lambda +{\left(-1+\alpha \right)}^{2}\beta^{3}\lambda^{2}-(-1+\alpha )\beta^{2}\lambda (2+(-1+\alpha )\lambda ))c_{n}^{2}}{{\left(-1+\alpha \right)}^{2}{\left(1+(-1+\alpha )\beta^{2}\lambda +\beta (\lambda -\alpha \lambda )\right)}^{2}})$$

**Corollary 2.** (i). $\frac{\partial p_{n}^{MA*}}{\partial c_{n}}>0,\frac{\partial p_{r}^{MA*}}{\partial c_{n}}>0$;

(ii). $\frac{\partial p_{r}^{MA*}}{\partial \beta }>0,\frac{\partial p_{r}^{MA*}}{\partial \lambda }<0$;

(iii). $\frac{\partial p_{n}^{MA*}}{\partial \alpha }>0,\frac{\partial p_{r}^{MA*}}{\partial \alpha }>0$.

Corollary 2 points out that under the agency model, the price at which the manufacturer sells the new and remanufactured product is positively related to the new product’s unit cost. The sales price of the remanufactured product is positively correlated with consumer preference for the remanufactured product, and negatively related to the sensitivity coefficient of recycling quantity to recycling price. This is consistent with scene MR. In scenario MA, the manufacturer has to pay a commission to the platform. The commission has a positive relationship with the sales price of both goods. This happens as a result of the manufacturer’s profit margins being reduced by increasing the fee. To improve profits, the manufacturer must raise prices.

### 4.3. Model ER

The e-commerce platform in Model ER is in charge of product recycling. The manufacturer creates new products and sells them to the e-commerce platform at wholesale prices. The products are recycled and reconstructed by the platform before being sold to customers. The decision sequence is: the manufacturer decides on the wholesale prices *w*_*n*_. The e-commerce platform then determines the selling prices *p*_*n*_ and *p*_*r*_. The profit functions f are as follows:

$$\pi_{M}^{ER}=(w_{n}-c_{n})D_{n}$$ (5)

$$\pi_{E}^{ER}=({p_{n}-w}_{n})D_{n}+p_{r}D_{r}-\frac{1}{\lambda }{D_{r}}^{2}$$ (6)

The equilibrium price and profit are as follows:

$$w_{n}^{ER*}=\frac{1}{2}(1+c_{n}),p_{n}^{ER*}=\frac{3+3\beta \lambda -\beta^{2}\lambda +c_{n}+\beta \lambda c_{n}}{4+4\beta \lambda },p_{r}^{ER*}=\frac{1}{4}\beta (\frac{3+2\beta \lambda }{1+\beta \lambda }+\frac{c_{n}}{1+\beta \lambda -\beta^{2}\lambda }),$$

$$\pi_{M}^{ER*}=\frac{\left(-1+c_{n})(-1-\beta \lambda +\beta^{2}\lambda +(1+\beta \lambda )c_{n}\right)}{8+8\beta \lambda -8\beta^{2}\lambda },$$

$$\pi_{E}^{ER*}=-\frac{1+2\beta \lambda +\beta^{2}\lambda^{2}-\beta^{4}\lambda^{2}+(-2-4\beta \lambda -2\beta^{2}(-2+\lambda )\lambda +4\beta^{3}\lambda^{2})c_{n}+{\left(1+\beta \lambda \right)}^{2}c_{n}^{2}}{16(1+\beta \lambda )(-1-\beta \lambda +\beta^{2}\lambda )}$$

**Corollary 3.** (i). $\frac{\partial w_{n}^{ER*}}{\partial c_{n}}>0,\frac{\partial p_{n}^{ER*}}{\partial c_{n}}>0,\frac{\partial p_{r}^{ER*}}{\partial c_{n}}>0$;

(ii). $\frac{\partial p_{n}^{ER*}}{\partial \beta }<0,\frac{\partial p_{r}^{ER*}}{\partial \beta }>0$;

(iii). $\frac{\partial p_{n}^{ER*}}{\partial \lambda }<0,\frac{\partial p_{r}^{ER*}}{\partial \lambda }<0.$

According to Corollary 3, the unit cost of manufacture of a new product has a positive correlation with all prices. This conclusion is consistent with Proposition 1. Consumer preferences for remanufactured products negatively affect the sales price of the new product and positively affect that of the remanufactured one. This is easily explained. Remanufactured items become more competitive and customers are more prepared to embrace them as their awareness of environmental issues grows. The remanufactured product’s price increases while the new product’s price decreases. The selling prices of both new and remanufactured goods are negatively correlated with the sensitivity coefficient of recycling quantity to recycling price. This is also consistent with the conclusion of Corollary 1. As *λ* increases, the platform’s recycling cost decreases, leading to a decrease in the selling price.

### 4.4. Model EA

Recycling under the model EA is the responsibility of the e-commerce platform. A commission is charged by the platform to the manufacturer, who creates new items and makes direct sales through it. The items are recycled and rebuilt by the platform before being sold to customers. The decision sequence is: the selling price of the new product (*p*_*n*_) is set by the manufacturer. The e-commerce platform also determines the remanufactured product’s selling price (*p*_*r*_), simultaneously. The profit functions are as follows:

$$\pi_{M}^{EA}=((1-\alpha )p_{n}-c_{n})D_{n}$$ (7)

$$\pi_{E}^{EA}=\alpha p_{n}D_{n}+p_{r}D_{r}-\frac{1}{\lambda }{D_{r}}^{2}$$ (8)

The equilibrium price and profit are as follows:

$$p_{n}^{EA*}=\frac{2(-1-\beta \lambda +\beta^{2}\lambda )((-1+\alpha )(-1+\beta )+c_{n})}{\left(-1+\alpha )(4-(5+\alpha )\beta^{2}\lambda +(1+\alpha )\beta^{3}\lambda +\beta (-2+4\lambda )\right)},p_{r}^{EA*}=\frac{\beta (-2-(1+\alpha )\beta \lambda +(1+\alpha )\beta^{2}\lambda )((-1+\alpha )(-1+\beta )+c_{n})}{\left(-1+\alpha )(4-(5+\alpha )\beta^{2}\lambda +(1+\alpha )\beta^{3}\lambda +\beta (-2+4\lambda )\right)},$$

$$\pi_{M}^{EA*}=\frac{(-1+\beta ){\left(2(-1+\alpha )(-1-\beta \lambda +\beta^{2}\lambda )+(-2-2\beta \lambda +(1+\alpha )\beta^{2}\lambda )c_{n}\right)}^{2}}{(-1+\alpha ){\left(4-(5+\alpha )\beta^{2}\lambda +(1+\alpha )\beta^{3}\lambda +\beta (-2+4\lambda )\right)}^{2}},$$

$$\pi_{E}^{EA*}=\frac{\left(-1-\beta \lambda +\beta^{2}\lambda )((-1+\alpha )(-1+\beta )+c_{n})((-1+\alpha )(-((-1+\beta )\beta^{2}\lambda )+\alpha^{2}(-1+\beta )\beta^{2}\lambda +\alpha (4+4\beta \lambda -4\beta^{2}\lambda ))-(\beta^{2}\lambda +\alpha^{2}\beta^{2}\lambda +2\alpha (-2-2\beta \lambda +\beta^{2}\lambda ))c_{n}\right)}{{\left(-1+\alpha \right)}^{2}(4-(5+\alpha )\beta^{2}\lambda +(1+\alpha )\beta^{3}\lambda +\beta (-2+4\lambda ))}$$

**Corollary 4.** (i). $\frac{\partial p_{n}^{EA*}}{\partial c_{n}}>0,\frac{\partial p_{r}^{EA*}}{\partial c_{n}}>0$;

(ii). $\frac{\partial p_{n}^{EA*}}{\partial \alpha }>0,\frac{\partial p_{r}^{EA*}}{\partial \alpha }>0$;

(iii). $\frac{\partial p_{n}^{EA*}}{\partial \lambda }<0,\frac{\partial p_{r}^{EA*}}{\partial \lambda }<0.$

As demonstrated by Corollary 4, the influence of the new product’s unit production cost and the product recycling quantity’s sensitivity coefficient to the recycling price on the equilibrium price in model EA are in line with the previous findings. That is, whether it is manufacturing costs or recycling costs, rising expenses will always result in higher pricing. Furthermore, there exists a positive correlation between commission rates and the sales price of both new and remanufactured goods. The product pricing structure on the market shifts as a new product’s price goes up with commissions. The platform will raise the sales price in order to stay competitive if it thinks that the market can handle higher pricing and that customers are still eager to purchase.

### 4.5. Pricing analysis

Through the equilibrium solutions of the wholesale and sales prices of new and remanufactured products under different scenarios, the following findings are reached.

**Proposition 1** The wholesale prices in the four scenarios have the following relationships:

(i). $w_{n}^{MR*}>w_{r}^{MR*};$
(ii). $w_{n}^{MR*}=w_{n}^{ER*}$.

Proposition 1 states that in the resale model, remanufactured items have a lower wholesale price than new products. The wholesale price of new products is not affected by the recycling entity. That is, regardless of whether the manufacturer or the platform is in charge of recycling and remanufacturing goods, the wholesale price of new products stays the same. The difference in wholesale prices is determined by production costs. Based on the hypothesis of the study, compared to remanufactured items, new products have a greater unit production cost, so there will be higher wholesale prices. The manufacturing and sales of remanufactured products are impacted by changes in the recycling system, while the production and selling of new products are unaffected, meaning that the wholesale costs of new products remain unchanged. To display the comparison between equilibrium prices more intuitively, we conducted numerical simulations. Numerical simulation methods have been widely used in the study of decision optimization. The numerical simulation in this article is based on the theoretical and practical basis of previous research, and the relevant parameters are assigned values [11,58].

**Fig 2:**
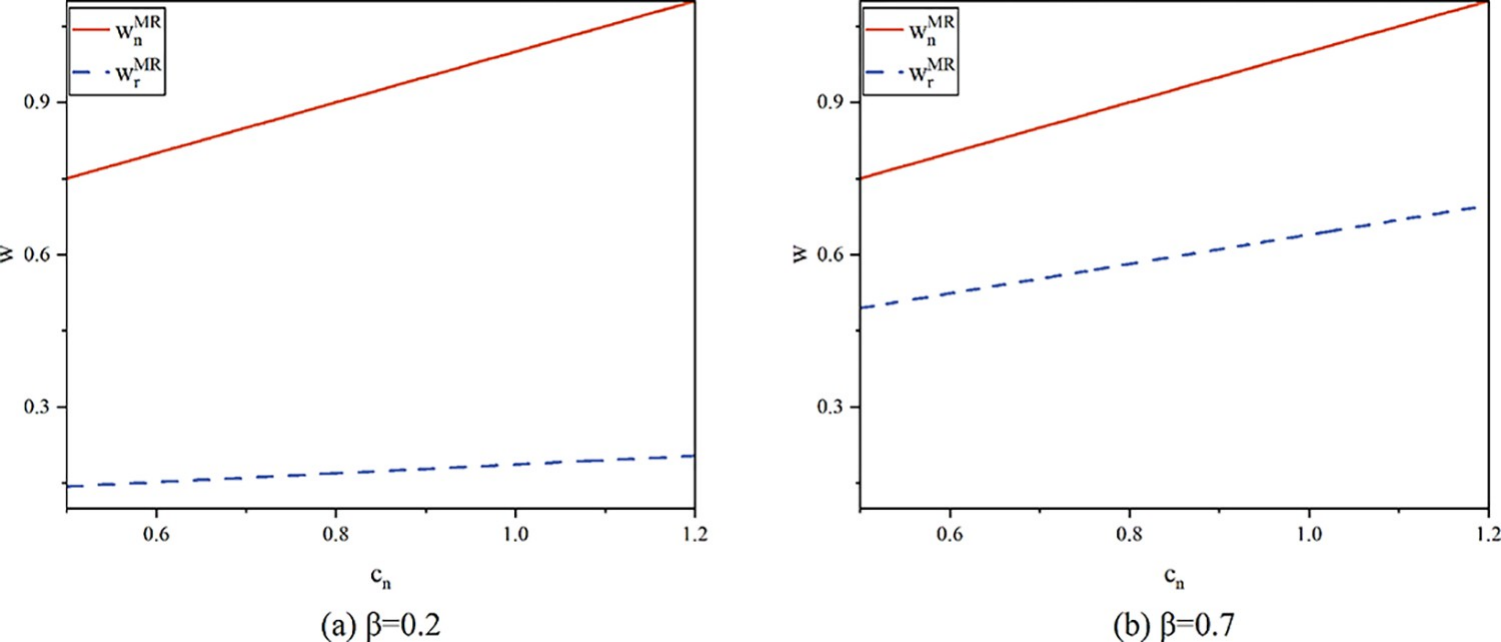
Wholesale prices of the new product and the remanufactured product in model MR (*λ* = 0.5).

Fig 2 illustrates how consumer desire for the remanufactured product causes the difference between the wholesale costs of the new product and the remanufactured product to close. This is consistent with cognition. The demand for the remanufactured product will rise due to the increased customer preference, which will also raise its price and reduce the difference between it and the new product.

**Proposition 2.** The selling prices in the four scenarios have the following relationships:

(i). $p_{n}^{MR*}>p_{n}^{MA*},when\;c_{n}<c_{n1};p_{n}^{ER*}>p_{n}^{EA*},when\;c_{n}<c_{n2}$;
(ii). $p_{n}^{MR*}>p_{n}^{ER*};p_{n}^{MA*}>p_{n}^{EA*},when\;c_{n}<c_{n3}$;
(iii). $p_{r}^{MR*}>p_{r}^{MA*},when\;c_{n}<c_{n4};p_{r}^{ER*}>p_{r}^{EA*},when\;c_{n}<c_{n5}$;
(iv). $p_{r}^{MR*}>p_{r}^{ER*},when\;c_{n}<c_{n6};p_{r}^{MA*}>p_{r}^{EA*},when\;c_{n}<c_{n7}$.

**Fig 3:**
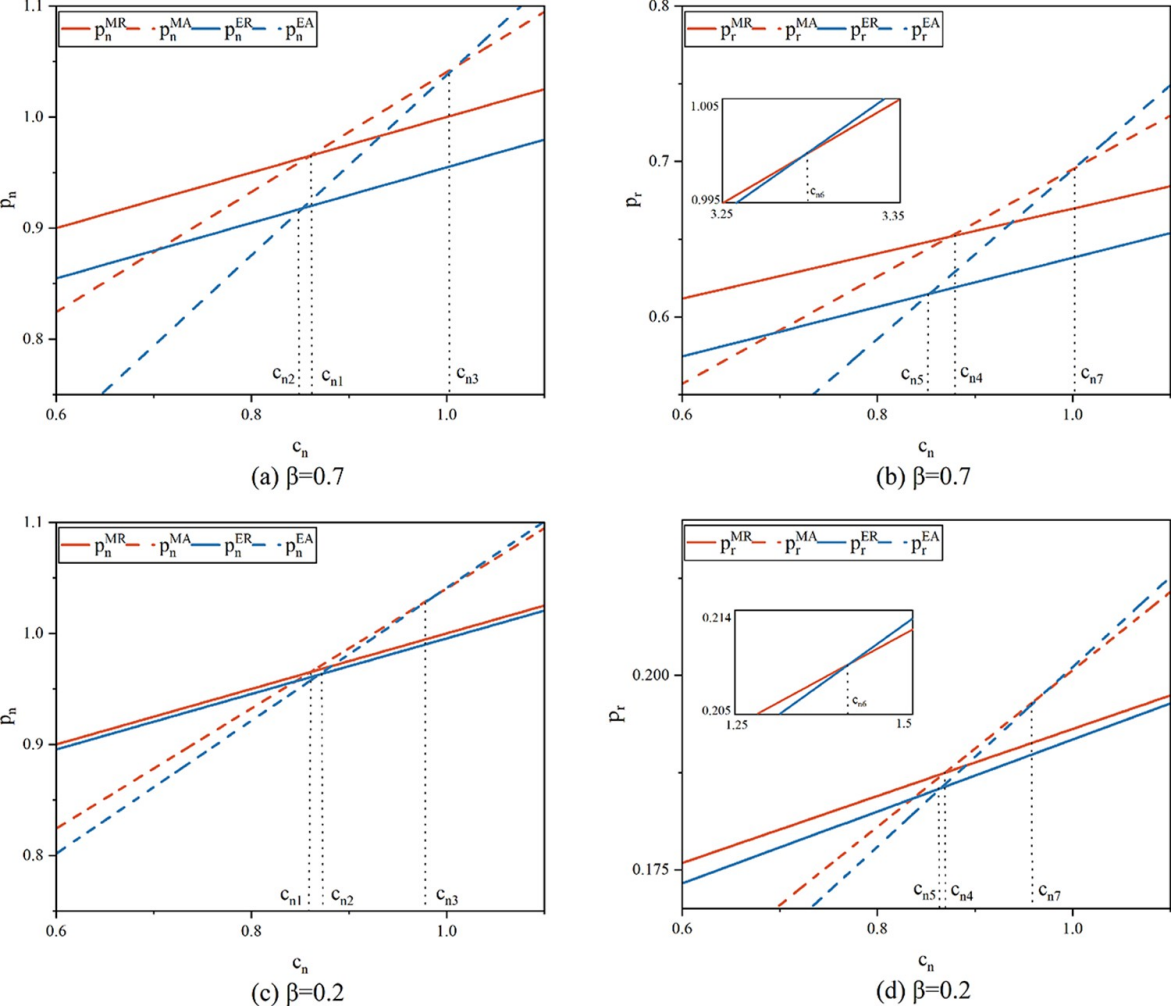
Sale prices of the new product and the remanufactured product (*λ* = 0.5, *α* = 0.075).

As seen in Fig 3, Proposition 2 indicates that the link between product sales prices and the new product’s unit manufacturing cost (*c*_*n*_) is significant. When *c*_*n*_ is small, the sales price of items under the resale model is higher than that of the agency model, according to a comparison of the prices of new and remanufactured goods under the same recycling structure. Comparing the prices under the same sales model, it is found that when *c*_*n*_ is small, the sales price of the product when recycled by the manufacturer is always greater than the price when recycled by the e-commerce platform. When new products are recycled by the manufacturer rather than the e-commerce platform, the sales price of the former is always higher under the resale model. The higher price under the resale model than the agency model can be explained by double marginalization. Under the resale model, the platform sets the retail price and sells the goods to the customer after the manufacturer sets the wholesale price. Both have profit maximization as their goal, and product pricing is subject to double marginalization, which is higher than in a model where the manufacturer sells at a direct price.

Whoever is in charge of recycling and remanufacturing does not affect the manufacturing and marketing process for the new product. However, a change in the recycling entity affects the remanufactured product. When the manufacturer carries out the recycling, the sales price of the new and remanufactured products are decided by the same subject, and when the platform carries out recycling, the manufacturer and the platform decide on the sales prices respectively. Since there is no way to observe the pricing of the other product, the price of the new product will be set in such a way as to avoid overpricing and losing the market. Simultaneous pricing provides better control over the sales price and market conditions. For the remanufactured goods, when *c*_*n*_ is low, the cost difference between the two products is small and the sales price of the product is the same as that of the new product. When *c*_*n*_ is high, the new product’s production costs are high. As a result, the manufacturer recycling model will customarily lower wholesale prices for the remanufactured product in order to maintain profit margins. As a result, the remanufactured product’s resale price will be lower than in the platform recycling scenario. Fig 3 illustrates how the price of both products rises in tandem with the new product’s unit cost of production. The price of a new product when recycled by the manufacturer under the resale model is always higher than the price via the recycling model on the e-commerce platform. Comparing Fig 3A–3D respectively, it can be seen that when consumers’ preference for the remanufactured product increases, for the new product, the sales price does not change much under the platform recycling model, but increases in the manufacturer recycling mode. For the remanufactured product, the reduction in *β* significantly reduces the sales price. This is due to reduced product preference leading to reduced product demand, thus sellers lower prices to attract consumers.

## 5. Recycling and channel strategies

In this section, we discuss the strategies of the supply chain members. After discussing the manufacturer’s and the e-commerce platform’s respective strategies, we go over win-win situations for both sides.

### 5.1. The manufacturer’s strategies

To study the manufacturer’s optimal sales model under different recycling models and the optimal recycling model under different sales models, we compare the manufacturer’s profit under different scenarios, and come up with the following conclusions.

**Proposition 3.** (i). $\pi_{M}^{MR*}>\pi_{M}^{MA*}$ when *λ* is low, $\pi_{M}^{MR*}<\pi_{M}^{MA*}$ when *λ* is high;

(ii).(1). $c_{n}<c_{n8},\pi_{M}^{ER*}>\pi_{M}^{EA*}$ when *α* is high, $\pi_{M}^{ER*}<\pi_{M}^{EA*}$ when *α* is low;(2). $c_{n}>c_{n8},\pi_{M}^{ER*}>\pi_{M}^{EA*}$ when *α* is low, $\pi_{M}^{ER*}<\pi_{M}^{EA*}$ when *α* is high.

Proposition 3 shows that in the model where the manufacturer is responsible for recycling, when *λ* is small the manufacturer chooses the resale model, otherwise the manufacturer chooses the agency model. In the model where the platform is responsible for recycling, when *c*_*n*_ is small, if the commission is high the manufacturer chooses the resale model, otherwise he chooses the agency model. In the product recycling framework where the manufacturer has the responsibility for recycling, the cost of recycling is high when *λ* is low and decreases as *λ* rises. The agency model requires the manufacturer to pay the commission. Due to cost structure considerations, the manufacturer chooses the agency model when the recycling cost is low to gain more profit. The conclusion of Proposition 3(ii) seems to contradict common sense, and we offer the following explanation. From (i) and (ii) of Proposition 1, when *c*_*n*_ is small, new products and remanufactured goods are offered at higher prices in the resale model; when *c*_*n*_ is large, new and remanufactured goods are offered at greater prices in the agency model. Meanwhile, the wholesale price increases as the unit cost of new products rises. So for the manufacturer, when new items have low unit production costs, the profit margin under the agency model becomes low as the commission rises, so he will choose the resale model. The manufacturer will select the agency model as *α* increases because the selling price of the product under the agency model is higher at this time and is positively correlated with the commission, which is necessary to obtain a larger profit margin when the unit cost of production of the new product is high.

**Fig 4:**
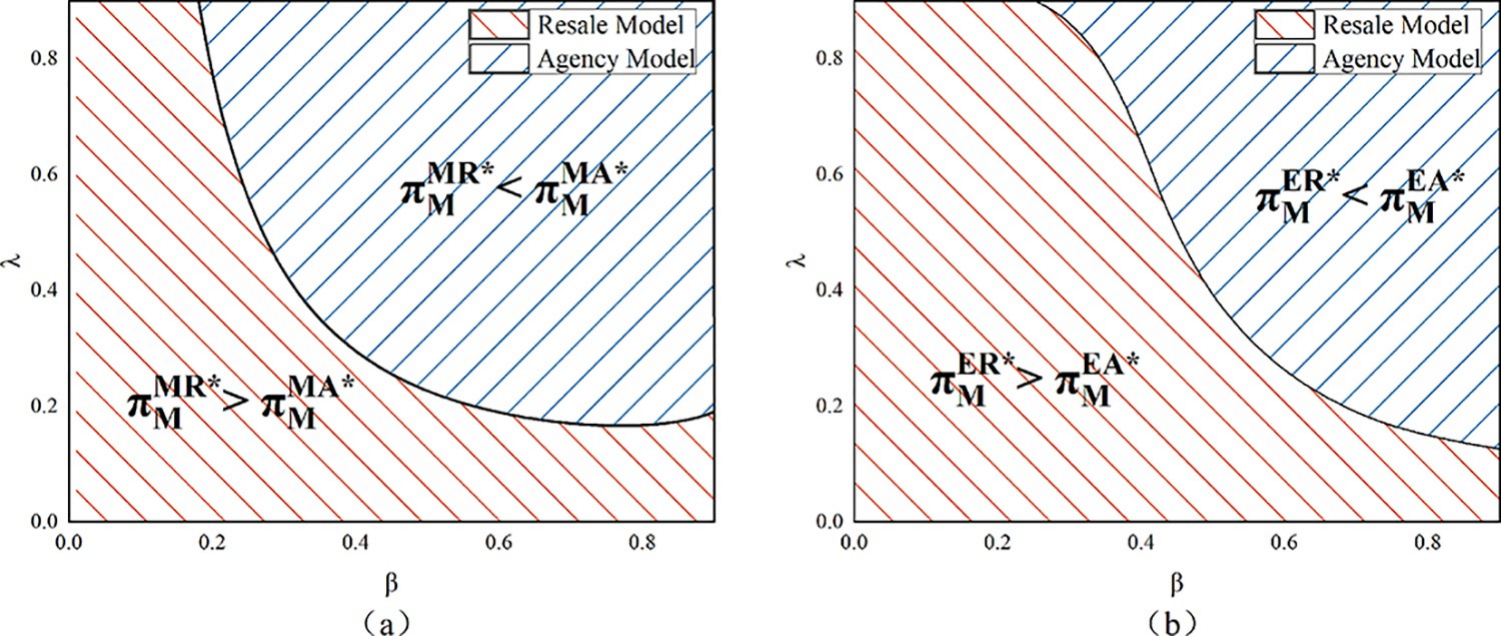
Manufacturer’s channel strategy in (a) the model with manufacturer recycling; (b) the model with e-commerce platform recycling (*c*_*n*_ = 0.9, *α* = 0.075).

We use numerical analysis to provide a graphic representation of the relationship between the manufacturer’s profit and customer demand for remanufactured products, as well as the sensitivity of recycling volume to recycling pricing. As shown in Fig 4, the manufacturer’s profit is higher in the resale model than in the agency model when the recycling quantity is less sensitive to the recycling price.

**Proposition 4.** $\pi_{M}^{MR*}>\pi_{M}^{ER*},\pi_{M}^{MA*}>\pi_{M}^{EA*}$.

**Fig 5:**
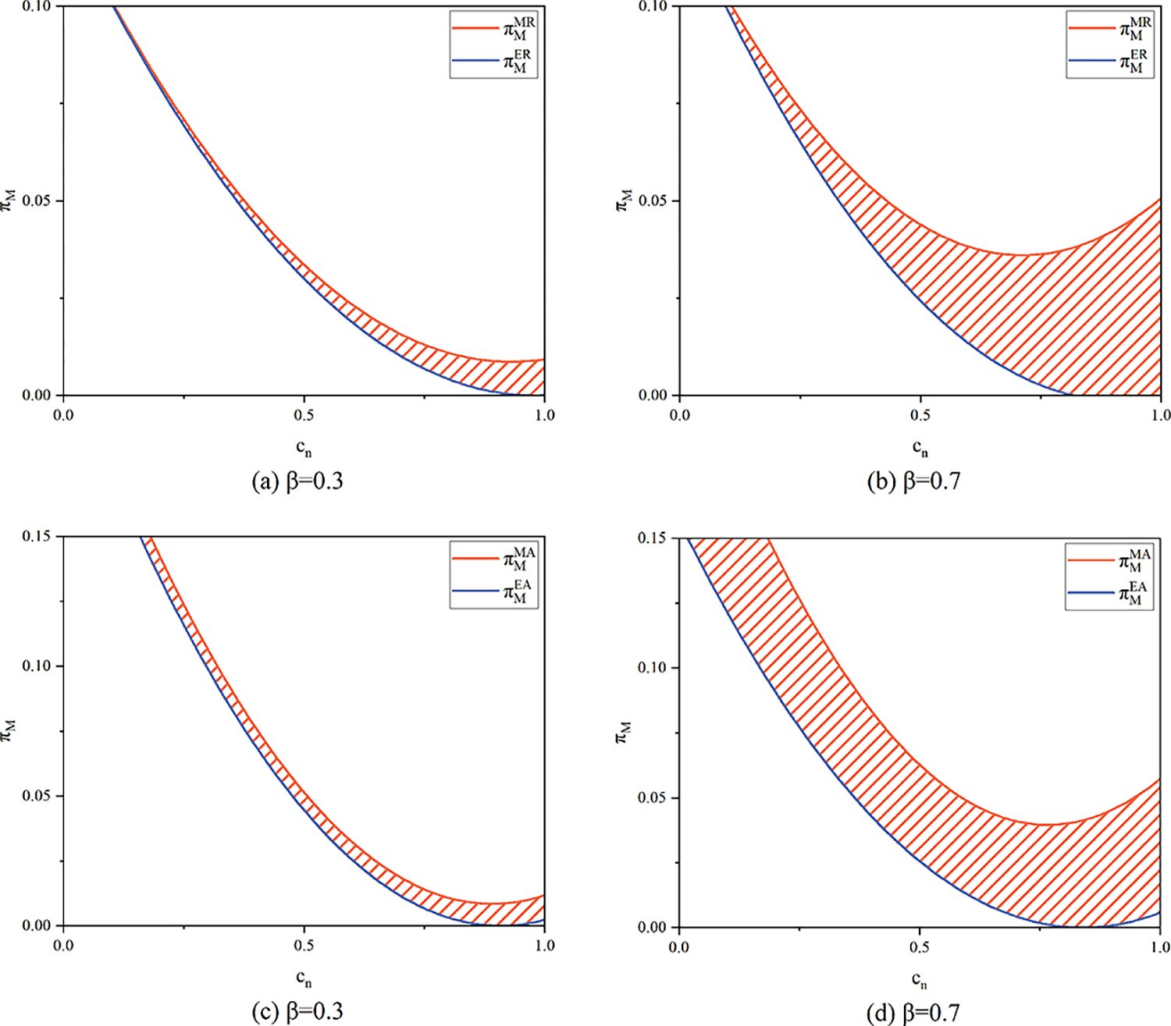
Comparison of manufacturer’s profits under manufacturer recycling and platform recycling models (*λ* = 0.5, *α* = 0.075).

Proposition 4 shows that in both the resale model and the agency mode, the manufacturer chooses to recycle himself, that is, given any sales model, the manufacturer is willing to be responsible for product recycling and remanufacturing himself. Under the resale model, the manufacturer doesn’t face consumers directly, making new products and selling them to the platform retailer at wholesale prices. If the manufacturer is responsible for recycling and remanufacturing and then wholesaling to the platform, he can better control the cost, realize the distribution of products and reduce the intensity of competition in the market. Under the agency mode, the manufacturer faces consumers directly. If he is also responsible for recycling, he can have better control over the entire CLSC process and can adjust his own pricing and profits. It can be seen that for the manufacturer, no matter what sales mode he is in, it is more profitable to take on the role of recycling and remanufacturing. As seen in Fig 5, the manufacturer’s profit difference (orange part in the figure) under the two recycling modes increases as consumers’ preference for remanufactured products increases. A rise in *β* increases the market demand for the remanufactured product, hence increasing the manufacturer’s profitability from recycling and producing the remanufactured product.

### 5.2. The E-commerce platform’s strategies

To study the platform’s optimal sales model under different recycling models and the optimal recycling model under different sales models, we compare the e-platform’s profit under various scenarios and come up with the following conclusions.

**Proposition 5.** (i). $\pi_{E}^{MR*}>\pi_{E}^{MA*}$ when *λ* is high, $\pi_{E}^{MR*}<\pi_{E}^{MA*}$ when *λ* is low;

(ii). (1). $c_{n}<c_{n9},\pi_{E}^{ER*}>\pi_{E}^{EA*}$ when *α* is low, $\pi_{E}^{ER*}<\pi_{E}^{EA*}$ when *α* is high; (2). $c_{n}>c_{n9},\pi_{E}^{ER*}>\pi_{E}^{EA*}$ when *α* is high, $\pi_{E}^{ER*}<\pi_{E}^{EA*}$ when *α* is low.

Proposition 5(i)shows that in the scenario when the recycling is conducted by the manufacturer, the platform chooses the resale model when the sensitivity of the recycling volume to the recycling price (*λ*) is high, and the platform chooses the agency model when *λ* is low. This is the opposite of the manufacturer’s choice in Proposition 3. A rise in *λ* represents a decrease in the cost of recycling. When the manufacturer’s recycling cost is low, the wholesale price is also lowered, and the platform can make more profit by wholesaling the product from the manufacturer and then re-selling it through the resale model. As the cost of recycling increases, the platform is unable to make higher profits through wholesale and then re-sale, so she prefers to adopt the agency model of charging a commission to the manufacturer.

**Fig 6:**
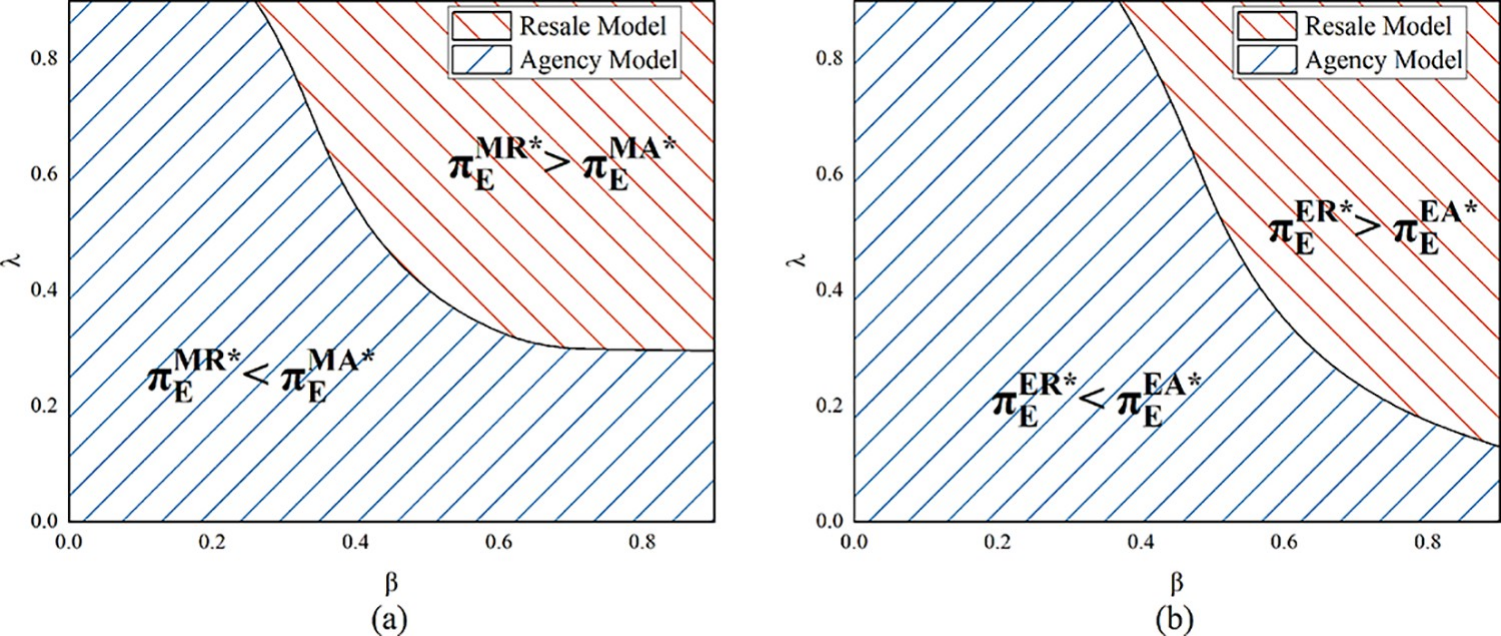
E-commerce platform’s channel strategy in (a) the model with manufacturer recycling; (b) the model with e-commerce platform recycling (*c*_*n*_ = 0.9,*α* = 0.075).

Proposition 5(ii) also concludes the opposite of Proposition 3. The platform’s decision is influenced by the new product’s unit manufacturing cost in the scenario where the platform is in charge of recycling. When *c*_*n*_ is small, the platform chooses resale when alpha is small, and when *c*_*n*_ is large, the platform chooses agency when alpha is small. When *c*_*n*_ is small, the price of the product is low, and the platform cannot make more profit from the agency model when *α* is low, so she chooses the resale model. When *c*_*n*_ is large, we know from Proposition 2 that the sales price under the agency model is greater. Meanwhile, as *α* increases, the price rises in both channels under the agent mode, and when *α* is too large, the excessively high price will have a dampening effect on demand and will have an impact on the platform’s profit, so she will shift to choose the resale mode. Similarly, we use numerical analysis to show in Fig 6 the impact of *β* and *λ* on platform profits. As Fig 6 illustrates, when the recycling amount is less sensitive to the recycling price, the manufacturer’s profit is larger in the resale model than in the agency model. Additionally, the manufacturer makes greater earnings in the resale model than in the agency model when consumer desire for the remanufactured product increases.

**Proposition 6.** $\pi_{E}^{MR*}<\pi_{E}^{ER*}$.

Proposition 6 suggests that in the resale model, the platform prefers to recycle by herself rather than the manufacturer. This finding suggests that the platform’s choice is opposite to that of the manufacturer (Proposition 4). The new product is manufactured by the manufacturer and then wholesaled to the platform under the resale model; the platform is not in charge of the new product’s manufacturing process. When the platform is in charge of recycling the product and remanufacturing and selling it, she has control over the remanufactured product and increases her competitiveness. The platform is thus able to make more profit. As stated in Proposition 6’s conclusion, the resale model does not present a situation in which both the manufacturer and the e-commerce platform benefit. The agency model’s platform profit comparison is complicated and comes from a numerical study.

**Fig 7:**
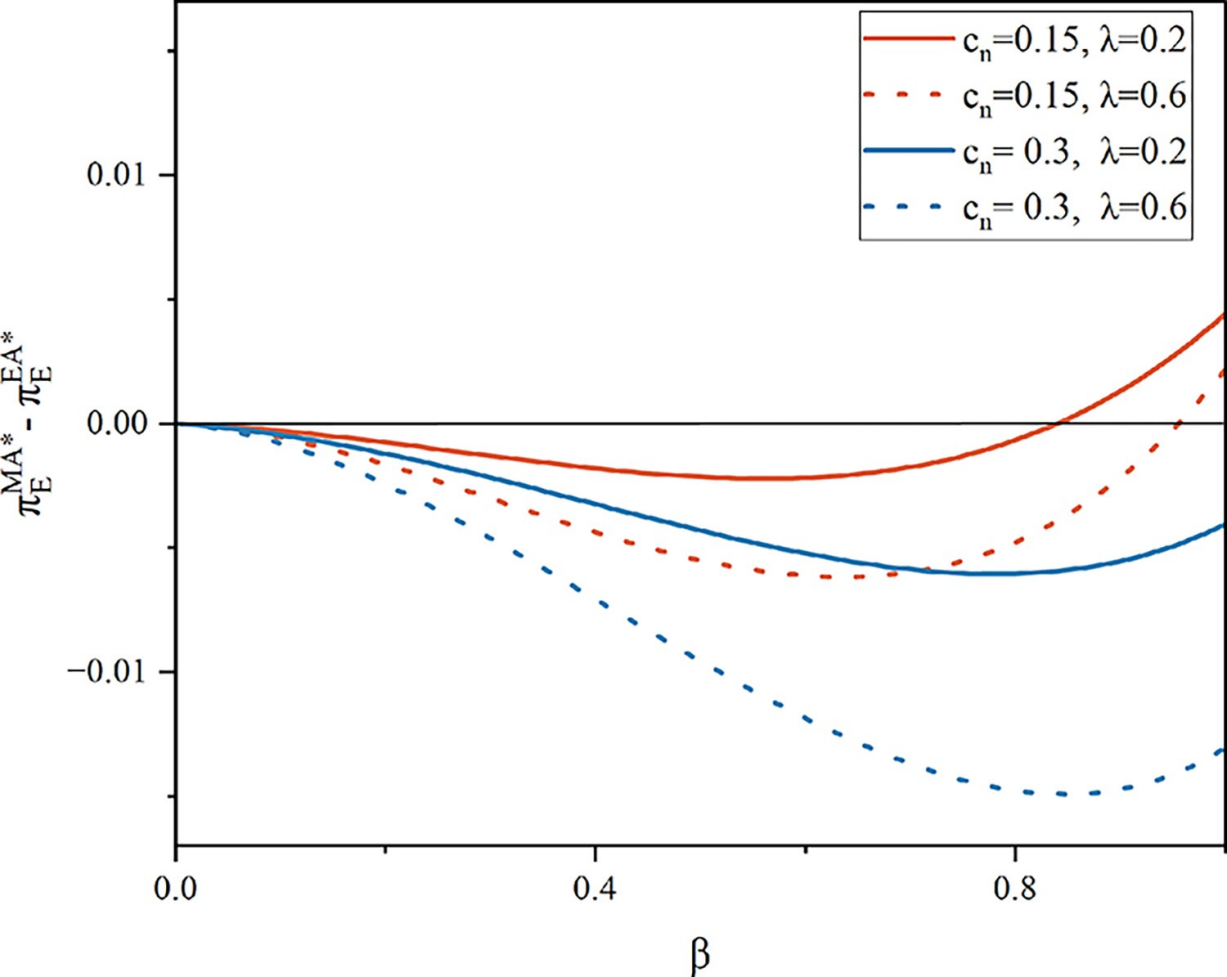
E-commerce platform’s profit in model MA and EA (*α* = 0.075).

As shown in Fig 7, in the agency model, when customer desire for the remanufactured product is low, the e-commerce platform chooses to perform product recycling herself. When the sensitivity of recycling volume to recycling price is low, the platform chooses the model in which the manufacturer conducts recycling. In addition, the cost of producing a new product rises per unit, the platform chooses the model where she conducts product recycling herself. Remanufactured products are in low demand when there is little customer desire for them. In the agency model, the platform has no sales revenue for new products, only commission revenue. Therefore, the platform’s profit margin is low if the manufacturer recycles the product and sells it to the platform in quantity. Similarly, when the unit production cost is high, the profit margin of selling the new product becomes small, and the commission that the platform can get becomes less. The platform prefers that the manufacturer shoulder the expense of recycling when *λ* is small because of its high cost.

**Fig 8:**
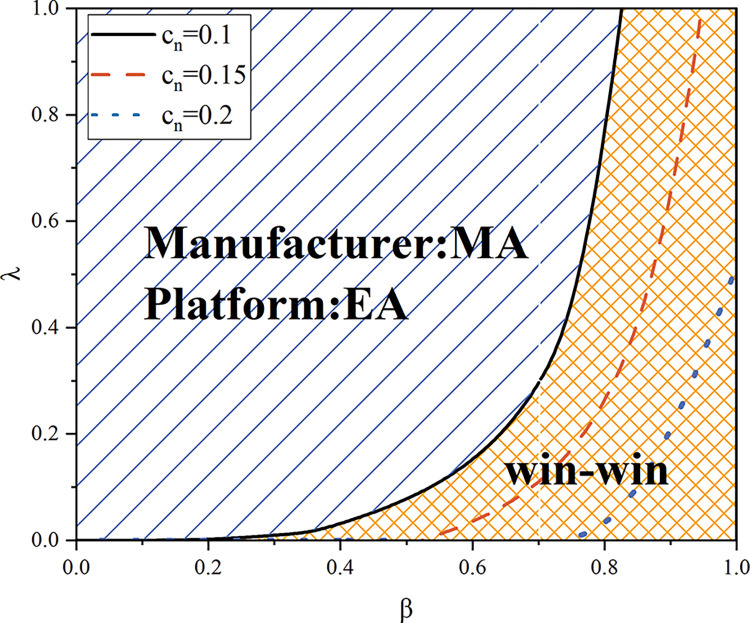
A win-win situation in the agency model (*α* = 0.075).

Because both the manufacturer and the e-commerce platform desire to recycle the goods themselves, Proposition 6 demonstrates that a win-win scenario cannot be achieved under the resale model. Under the agency model, as can be seen in Fig 8, when *β* is high, a win-win scenario can be achieved. When *β* is high, as Fig 7 illustrates, the e-commerce platform also wants to carry out product recycling and sales of remanufactured products by the manufacturer, so a win-win situation is achieved at this point. When *c*_*n*_ rises, the win-win space becomes smaller (the orange area in the figure shrinks from the black to the blue line). The same effect occurs when the sensitivity coefficient of the recycling quantity to the recycling price becomes larger. As analyzed earlier, an increase in both makes the platform more willing to choose the model of having herself carry out the recycling, which is the opposite of the manufacturer’s choice, so it will lead to a smaller win-win scope.

## 6. Consumer surplus

In this section, we analyze consumer surplus under different scenarios. In green supply chains, consumer surplus is an important indicator for evaluating corporate social responsibility and sustainable development. Consumer surplus represents the residual utility of consumers after purchasing a product, and is usually calculated through the maximum product sales price acceptable to consumers and the actual sales price [60,61].

The consumer surplus can be expressed as *CS* = *CS*_*n*_+*CS*_*r*_, where ${CS}_{n}=\frac{1}{2}(\hat{p_{n}}-p_{n}^{*}),{CS}_{r}=\frac{1}{2}(\hat{p_{r}}-p_{r}^{*})$. Through the demand functions *D*_*n*_ and *D*_*r*_, the maximum acceptable prices $\hat{p_{n}}$ and $\hat{p_{r}}$ of consumers for the new and the remanufactured products are: $\hat{p_{n}}=1-\beta +p_{r}$ and $\hat{p_{r}}=\beta p_{n}$.

So the consumer surpluses in different models are as follows:

$${CS}^{MR*}=-\frac{\left((\beta -1)({\left(-2\beta^{2}\lambda +2\beta \lambda +1\right)}^{2}+(4\beta^{3}\lambda^{2}+4\beta^{2}\lambda^{2}+4\beta \lambda +1)c_{n}^{2}+(8\beta^{3}\lambda^{2}+4\beta^{2}\lambda (1-2\lambda )-8\beta \lambda -2)c_{n})\right)}{32{\left(-2\beta^{2}\lambda +2\beta \lambda +1\right)}^{2}}$$

$${CS}^{MA*}=\frac{\left(-1+\beta )(-\beta^{3}\lambda^{2}c_{n}^{2}-\frac{1}{{\left(-1+\alpha \right)}^{2}}{\left((-1+\alpha )(1+(-1+\alpha )\beta^{2}\lambda +\beta (\lambda -\alpha \lambda ))+(1+\beta (\lambda -\alpha \lambda ))c_{n}\right)}^{2}\right)}{8{\left(1+(-1+\alpha )\beta^{2}\lambda +\beta (\lambda -\alpha \lambda )\right)}^{2}}$$

$${CS}^{ER*}=-(\begin{array}{c} ((-1+\beta )({\left(1+\beta \lambda -\beta^{2}\lambda \right)}^{2}(1+2\beta \lambda +\beta^{2}\lambda^{2}+\beta^{3}\lambda^{2})-2(1+4\beta \lambda +4\beta^{3}(-1+\lambda )\lambda^{2}\\ +\beta^{4}(-5+\lambda )\lambda^{3}+\beta^{6}\lambda^{4}+\beta^{2}\lambda (-1+6\lambda )+\beta^{5}(\lambda^{3}-2\lambda^{4}))c_{n}+{\left(1+\beta \lambda \right)}^{2}(1+2\beta \lambda \\ +\beta^{2}\lambda^{2}+\beta^{3}\lambda^{2})c_{n}^{2})) \end{array}/\left(32{\left(1+\beta \lambda \right)}^{2}{\left(1+\beta \lambda -\beta^{2}\lambda \right)}^{2}\right))$$

$${CS}^{EA*}=-(\begin{array}{c} ((-1+\beta )({\left(-1+\alpha \right)}^{2}(4+8\beta \lambda +4\beta^{2}(-2+\lambda )\lambda \\ +(-7-2\alpha +\alpha^{2})\beta^{3}\lambda^{2}-2(-1-2\alpha +\alpha^{2})\beta^{4}\lambda^{2}\\ +{\left(-1+\alpha \right)}^{2}\beta^{5}\lambda^{2})+2(-1+\alpha )(4+8\beta \lambda -(7+\alpha^{2})\beta^{3}\lambda^{2}\\ +(3+\alpha^{2})\beta^{4}\lambda^{2}+2\beta^{2}\lambda (-3-\alpha +2\lambda ))c_{n}+(4+8\beta \lambda \\ +(-3-6\alpha +\alpha^{2})\beta^{3}\lambda^{2}+{\left(1+\alpha \right)}^{2}\beta^{4}\lambda^{2}+4\beta^{2}\lambda (-1-\alpha +\lambda ))c_{n}^{2})) \end{array}/\begin{array}{c} (2{\left(-1+\alpha \right)}^{2}\\ {\left(4-(5+\alpha )\beta^{2}\lambda +(1+\alpha )\beta^{3}\lambda +\beta (-2+4\lambda )\right)}^{2}) \end{array})$$

**Fig 9:**
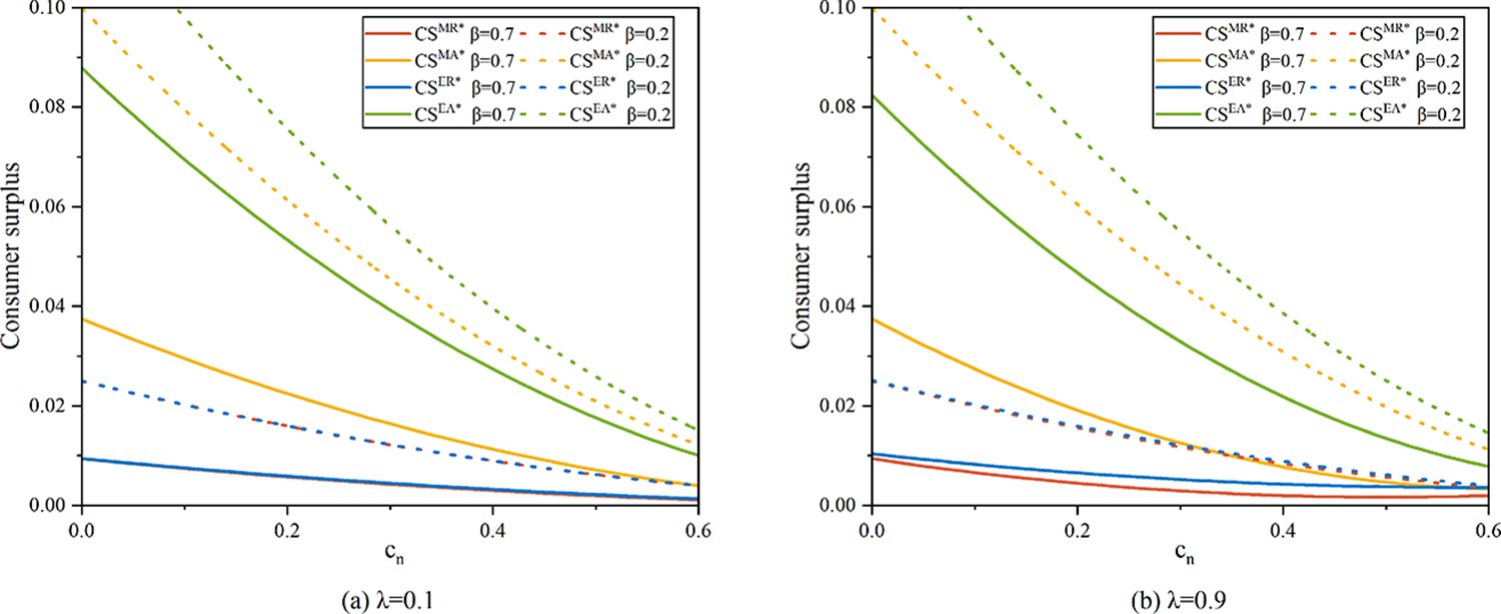
Consumer surplus in different scenarios (*α* = 0.075).

Through numerical analysis, we compare consumer surplus in different models. Of the four models, model EA has the largest consumer surplus, as seen in Fig 9. It can be seen from Proposition 2 that when the unit production cost of a new product (*c*_*n*_) is low, the product sales price under model EA is the lowest, so consumers can obtain higher surplus value. In addition, the agency model yields a higher consumer surplus than the resale model, regardless of who is the recycling entity. Because double marginalization is serious under the resale model, it affects consumer utility. Comparing the solid and dotted lines in Fig 9, it is evident that consumer surplus rises as customers’ inclination for remanufactured goods declines. This is because when consumers’ willingness to buy decreases, demand will decrease. In order to increase demand, the manufacturer or the platform will lower product sales prices, resulting in consumers receiving more residual value. Comparing Fig 9A and 9B, an increase in the sensitivity of recycling volume to recycling price will increase consumer surplus. As mentioned earlier,*λ* reflects the cost of recycling, and as *λ* rises, the cost of recycling becomes lower. Lower costs will cause product sellers to lower their selling prices, resulting in higher residual value for consumers.

## 7. Conclusions

In this study, a closed-loop e-commerce supply chain is established, and the issue of selecting sales and recycling models for the manufacturer and e-commerce platform is examined. We investigate the resale and agency models in e-commerce, as well as two recycling models wherein the responsibility for recycling and selling remanufactured products lies with the manufacturer and the e-commerce platform, respectively. By merging the models, we create four research scenarios. We analyzed the equilibrium solutions under each model and examined the strategies of manufacturers and e-commerce platforms. The conclusions and management takeaways are listed below.

### 7.1. Main findings

This study establishes an e-commerce closed-loop supply chain research model based on existing research, supplements the research conclusions in this field, and has theoretical significance.

Savaskan et al. [5] earlier studied the problem of different recycling entities in the closed-loop supply chain. Comparing the models of manufacturer recycling, retailer recycling and third-party recycling respectively, they found that under decentralized decision-making, the optimal recycling method for the manufacturer is retailer recycling. By establishing a combination of different sales models and recycling models in a closed-loop supply chain in the context of e-commerce, and comparing equilibrium solutions in different scenarios, this study not only obtained the manufacturer’s optimal strategy, but also obtained the e-commerce platform’s strategy selection.

First, for the manufacturer, in the scenario when recycling is his responsibility, when there is little customer interest in remanufactured goods or when there is little correlation between the price and quantity of recycling, the resale model is selected. In the scenario when recycling is the platform’s responsibility, when the unit production cost is small, the manufacturer with large commissions will choose the resale model, otherwise, he will choose the agency model. Cost is clearly a major factor in the manufacturer’s choice of sales strategy under various recycling scenarios. Within a given sales model, the manufacturer has the same recycling model choices. Specifically, when it comes to agency or resale modes, the manufacturer will always decide to recycle on his own. In addition, whatever the recycling strategy, the manufacturer’s profit under the agency model tends to surpass that under the resale model as consumers’ interest in remanufactured goods increases.

Secondly, for the platform, in the model where the manufacturer is responsible for recycling, in situations when recycling volume and recycling price are highly correlated, she usually selects the resale model and vice versa. The choice of platform is influenced by the unit production cost of new goods under the model where the platform is in charge of recycling products. In instances when costs are minimal, the platform selects an agency as commission levels rise. The platform selects resale when the cost is high and the commission rises. Under a given sales model, the e-commerce platform prefers to handle recycling herself rather than the manufacturer in the resale model. Since the strategy differs from the manufacturer’s, the resale model cannot result in a win-win scenario. When customers have little interest in remanufactured goods, the e-commerce platform opts to recycle the products herself under the agency model. The platform favors the manufacturer’s recycling model when the amount of recycling is less sensitive to the price of recycling. In addition, the platform chooses to recycle herself when the cost of producing new goods rises per unit.

Since the decision-making goals of both parties in the game are to maximize their own interests, conflicts will inevitably exist when choosing between different modes. Zhang et al. [31] showed in their study that when there is no government-funded coordination, the remanufacturing model that is the responsibility of the manufacturer will harm the retailer’s profits. Quan et al. [53] compared the models in which the manufacturer is responsible for recycling and the retailer is responsible for recycling and found that in most cases, the manufacturer and retailer prefer to be responsible for recycling themselves. In addition, they studied the conditions under which both parties can reach an agreement (i.e., a win-win situation), that is, when the market potential of the two periods meets certain conditions, a win-win situation can be achieved. Our study also analyzes the conditions for achieving a win-win situation for supply chain members by comparing the equilibrium strategies of the manufacturer and the e-commerce platform.

By examining the strategies used by supply chain participants, we discover that in the agency model, the platform will choose a recycling model within a specific range, which is consistent with the manufacturer’s choice, so it can achieve a win-win situation. Customers’ strong desire for the remanufactured product or a low sensitivity of recycling volume to recycling pricing is the prerequisite. A larger win-win scope can be achieved when the unit production cost of new products is low. Since both parties’ choices in the reselling model are opposing ones, a win-win scenario is not possible.

In the study of the closed-loop supply chain, consumer surplus has been fully discussed by scholars. Mu et al. [12] discussed the consumer surplus problem in the green supply chain and analyzed the impact of green product segmentation strategies. Jia and Li [57] discussed the consumer surplus under four distribution models in the closed-loop supply chain and found that there were different results under different order fulfillment costs and platform commission rates. In the trade-in problem, different models have different impacts on consumer surplus. If the waiting cost of consumers is high, the cooperation model between the enterprise and the platform can bring a higher consumer surplus than the self-built model [3].

Our study compares consumer surplus under different combinations of recycling and sales models in closed-loop supply management in the context of e-commerce and finds that consumer surplus is highest under the agency model where the platform is responsible for recycling, at which point double marginalization is alleviated. In addition, the reduction in consumer preference for remanufactured products and the reduction in recycling costs of remanufactured products will lead to an increase in consumer surplus.

### 7.2. Managerial implecations

Through the analysis of the theoretical model, we provide some management implications for supply chain members.

Manufacturers in closed-loop supply chains need to fully consider consumer preferences for remanufactured products when making decisions. From the conclusion, we see that consumer preferences for remanufactured products will affect manufacturers’ profits under different recycling and sales models. Categories with high consumer acceptance of remanufactured products are usually those with higher value, shorter life cycles, longer service life, and relatively affordable prices. Acceptance of remanufacturing is generally lower for products that require a high degree of hygiene, safety and performance, and for those that are closely related to individual tastes and preferences. Based on this conclusion, manufacturers can decide based on product category when choosing a sales mode. For a given recycling model, the manufacturers can prioritize the resale sales model for remanufactured products with low consumer preference, such as medical devices. Furthermore, given the sales model, manufacturers should give priority to recycling products themselves. Because from conclusion we see that higher profits can be achieved by recycling by the manufacturer himself. In other words, manufacturers should proactively pursue the important role of recyclers in CLSC.

There will be distinct equilibria since the manufacturer’s and the platform’s best options differ. Since the optimal choices of platforms and manufacturers are not entirely consistent, this means that when one party has more say, the interests of the other party may be harmed. This also means that different equilibrium situations will occur under different power structures. When the platform can make relevant decisions, as mentioned earlier, consumers’ preference of the remanufactured products should be taken into consideration. For example, under the agency model, for the remanufactured products that aren’t welcomed by consumers, the platform can proactively provide recycling services and act as a recycler to obtain higher profits. When the manufacturer is the formulation of decision-making, the platform can improve the profitability in the corresponding model, such as by increasing commissions.

Since a win-win situation can only be achieved under certain conditions, if the e-commerce platform and the manufacturer only make decisions from their own perspectives, no matter what kind of power structure they are under, one party may be harmed. Such a cooperation model is bound to not be a stable structure and is not conducive to the long-term stable development of the supply chain. When a win-win situation cannot be reached, both supply chain members should strengthen communication, reduce conflicts, and avoid damage to the profits. In a given sales model, the manufacturer hopes to recycle by itself, but the platform has different optimal choices in the resale mode. Therefore, to meet the optimal conditions, manufacturers should give priority to choosing an agency model to promote the realization of a win-win. Furthermore, compared to the resale model, the agency model has a larger consumer surplus. In order to assume corporate responsibility and improve social welfare, supply chain members should also work together to achieve a win-win situation and bring more utility to consumers under the agency model.

This study also has certain implications for government management decisions. On the one hand, given the conflicts that may arise in CLSC, the government should assume the responsibility for coordination. The government can establish a market coordination mechanism to improve the communication efficiency of supply chain members. When there is a decision-making conflict between the two sides of the supply chain, the government can coordinate and maintain the stability of the closed-loop supply chain. On the other hand, from the perspective of environmental sustainability and consumer welfare, the government should improve the public’s environmental awareness and encourage consumers to participate in environmental protection practices through publicity and science popularization. To avoid the reduction of utility caused by the increase in consumers’ environmental awareness, the government should regulate the price of remanufactured products. At the same time, to encourage supply chain members to participate in environmental protection practices, appropriate subsidies can be provided to reduce the cost of recycling and remanufacturing for supply chain members to increase their enthusiasm.

For consumers, we see from the research results that as consumers increase their preference for remanufactured products, consumer surplus will decrease. On the one hand, consumers should increase their awareness of environmental protection and voluntarily participate in environmentally sustainable practices. On the other hand, to protect their rights and interests, consumers should actively participate in the government’s supervision of remanufactured product pricing and jointly promote the good operation of the closed-loop supply chain.

### 7.3. Limitations

This study seeks to address the issue of channel and recycling mode selection in the e-commerce closed-loop supply chain, but there are still some limitations. For example, remanufacturing and recycling are the responsibility of the same supply chain member in this study. Future research may consider situations where remanufacturing and recycling responsibilities are separated, such as the presence of third-party corporate recycling, and discuss more scenarios. Further, scenarios of government subsidies or regulations can also be considered to solve more practical problems.

## Supporting information

S1 Appendix Proofs of propositions. (DOCX)
