# Supplementary material for: Remanufacturing and channel strategies in e-commerce closed-loop supply chain
Source: PLoS One. 2024 May 16;19(5):e0303447. doi: 10.1371/journal.pone.0303447 (PMC11098343; doi:10.1371/journal.pone.0303447)
Supplement: S1 Appendix — (DOCX) [file pone.0303447.s001.docx]

**Appendix: Proofs of propositions**

**Proof of Corollary 1.** In Model MR, we have the profits$\pi_{M}^{MR}$and $\pi_{E}^{MR}$.As the leader of the Stackelberg game, the manufacturer first makes the wholesale prices decision, and the e-commerce platform makes the resale prices decision after observing the wholesale prices. We adopt backward induction to solve the problem. First, we find the resale prices that maximize the profit of the e-commerce platform. The Hessian matrix of $\pi_{E}^{MR}$ on ($p_{n}, p_{r}$) is $H=\left[ \begin{matrix} \frac{\partial^{2}\pi_{E}^{MR}}{\partial{p_{n}}^{2}} & \frac{\partial^{2}\pi_{E}^{MR}}{\partial p_{n}p_{r}} \\ \frac{\partial^{2}\pi_{E}^{MR}}{\partial{p_{r}p}_{n}} & \frac{\partial^{2}\pi_{E}^{MR}}{\partial{p_{r}}^{2}} \end{matrix} \right]$=$\left[ \begin{matrix} \frac{2}{-1+\beta} & -\frac{2}{-1+\beta} \\ -\frac{2}{-1+\beta} & \frac{2}{(-1+\beta)\beta} \end{matrix} \right]$, which is negatively definite. Solving the first-order conditions $\frac{\partial\pi_{E}^{MR}}{\partial p_{n}}=0$and $\frac{\partial\pi_{E}^{MR}}{\partial p_{r}}=0$for ($p_{n}^{*}, p_{r}^{*}$)= ($\frac{1}{2}\left( 1+w_{n} \right),\frac{1}{2}(\beta+w_{r})$). Then substitute ($p_{n}^{*}, p_{r}^{*}$) into $\pi_{M}^{MR}$.We can get $\pi_{M}^{MR}\left( w_{n}, w_{r} \right)=\frac{1}{4{(-1+\beta)}^{2}\beta^{2}\lambda}(\beta^{2}(-1+2(-1+\beta)\lambda)w_{n}^{2}-2(-1+\beta)\beta^{2}\lambda c_{n}(-1+\beta+w_{n}-w_{r})+(-1+2(-1+\beta)\beta\lambda)w_{r}^{2}+2\beta w_{n}({(-1+\beta)}^{2}\beta\lambda+(1-2(-1+\beta)\beta\lambda)w_{r}))$. Next, we have the Hessian matrix $\pi_{M}^{MR}\left( w_{n}, w_{r} \right)$on ($w_{n}, w_{r}$) is$H=\left[ \begin{matrix} \frac{\partial^{2}\pi_{M}^{MR}}{\partial{w_{n}}^{2}} & \frac{\partial^{2}\pi_{M}^{MR}}{\partial w_{n}w_{r}} \\ \frac{\partial^{2}\pi_{M}^{MR}}{\partial{w_{r}w}_{n}} & \frac{\partial^{2}\pi_{M}^{MR}}{\partial{w_{r}}^{2}} \end{matrix} \right]$=$\left[ \begin{matrix} \frac{1}{-1+\beta}-\frac{1}{2{(-1+\beta)}^{2}\lambda} & \frac{1-2(-1+\beta)\beta\lambda}{2{(-1+\beta)}^{2}\beta\lambda} \\ \frac{1-2(-1+\beta)\beta\lambda}{2{(-1+\beta)}^{2}\beta\lambda} & \frac{-1+2(-1+\beta)\beta\lambda}{2{(-1+\beta)}^{2}\beta^{2}\lambda} \end{matrix} \right]$, which is negatively definite. Solving the first-order conditions $\frac{\partial\pi_{M}^{MR}}{\partial w_{n}}=0$and $\frac{\partial\pi_{M}^{MR}}{\partial w_{r}}=0$for ($w_{n}^{*}, w_{r}^{*}$). Thus the optimal wholesale prices are $w_{n}^{*}=\frac{1}{2}\left( 1+c_{n} \right)$,$w_{r}^{MR*}=\frac{\beta\left( 1+2\beta\lambda-2\beta^{2}\lambda+c_{n} \right)}{2+4\beta\lambda-4\beta^{2}\lambda}$.Bringing($w_{n}^{*}, w_{r}^{*}$) into ($p_{n}^{*}, p_{r}^{*}$) can find the optimal resale price. The optimal sales prices are $p_{n}^{MR*}=\frac{1}{4}\left( 3+c_{n} \right), p_{r}^{MR*}=\frac{\beta\left( 3+6\beta\lambda-6\beta^{2}\lambda+c_{n} \right)}{4+8\beta\lambda-8\beta^{2}\lambda}$.Finally, the equilibrium profits can be obtained as:$\pi_{M}^{MR*}=\frac{-1-2\beta\lambda+2\beta^{2}\lambda+\left( 2+4\beta\lambda-4\beta^{2}\lambda\right)c_{n}-\left( 1+2\beta\lambda\right)c_{n}^{2}}{8\left( -1-2\beta\lambda+2\beta^{2}\lambda\right)}$,$\pi_{E}^{MR*}=\frac{1}{16}(1-2c_{n}+\frac{\left( 1+4\beta\lambda+4\beta^{2}\left( -1+\lambda\right)\lambda-4\beta^{3}\lambda^{2} \right)c_{n}^{2}}{\left( 1+2\beta\lambda-2\beta^{2}\lambda\right)^{2}})$.

According to the equilibrium solutions, we have:

(i) $\frac{\partial w_{n}^{MR*}}{\partial c_{n}}=\frac{1}{2}>0$,$\frac{\partial w_{r}^{MR*}}{\partial c_{n}}=\frac{\beta}{2-4(-1+\beta)\beta\lambda}>0$,$\frac{\partial p_{n}^{MR*}}{\partial c_{n}}=\frac{1}{4}>0, \frac{\partial p_{r}^{MR*}}{\partial c_{n}}=\frac{\beta}{4-8(-1+\beta)\beta\lambda}>0$;

(ii). $\frac{\partial w_{r}^{MR*}}{\partial\beta}=\frac{1}{2}\left( 1+\frac{\left( 1+2\beta^{2}\lambda\right)c_{n}}{\left( 1+2\beta\lambda-2\beta^{2}\lambda\right)^{2}} \right)>0,\frac{\partial p_{r}^{MR*}}{\partial\beta}=\frac{1}{4}(3+\frac{(1+2\beta^{2}\lambda)c_{n}}{{(1+2\beta\lambda-2\beta^{2}\lambda)}^{2}})>0$;

(iii). $\frac{\partial w_{r}^{MR*}}{\partial\lambda}=\frac{(-1+\beta)\beta^{2}c_{n}}{{(1+2\beta\lambda-2\beta^{2}\lambda)}^{2}}<0,\frac{\partial p_{r}^{MR*}}{\partial\lambda}=\frac{(-1+\beta)\beta^{2}c_{n}}{2{(1+2\beta\lambda-2\beta^{2}\lambda)}^{2}}<0$. □

**Proof of Corollary 2.** In Model MA, we have the profits$\pi_{M}^{MA}$and $\pi_{E}^{MA}$.In this model, the manufacturer does not make pricing decisions, only the e-commerce platform needs to decide the resale price. The Hessian matrix of $\pi_{E}^{MA}$ on ($p_{n}, p_{r}$) is $H=\left[ \begin{matrix} \frac{\partial^{2}\pi_{E}^{MA}}{\partial{p_{n}}^{2}} & \frac{\partial^{2}\pi_{E}^{MA}}{\partial p_{n}p_{r}} \\ \frac{\partial^{2}\pi_{E}^{MA}}{\partial{p_{r}p}_{n}} & \frac{\partial^{2}\pi_{E}^{MA}}{\partial{p_{r}}^{2}} \end{matrix} \right]$=$\left[ \begin{matrix} -\frac{2(1+(-1+\alpha)(-1+\beta)\lambda)}{{(-1+\beta)}^{2}\lambda} & \frac{2(1+(-1+\alpha)\beta^{2}\lambda+\beta(\lambda-\alpha\lambda))}{{(-1+\beta)}^{2}\beta\lambda} \\ \frac{2(1+(-1+\alpha)\beta^{2}\lambda+\beta(\lambda-\alpha\lambda))}{{(-1+\beta)}^{2}\beta\lambda} & -\frac{2(1+(-1+\alpha)\beta^{2}\lambda+\beta(\lambda-\alpha\lambda))}{{(-1+\beta)}^{2}\beta^{2}\lambda} \end{matrix} \right]$, which is negatively definite. Solving $\frac{\partial\pi_{E}^{MA}}{\partial p_{n}}=0$and $\frac{\partial\pi_{E}^{MA}}{\partial p_{r}}=0$for ($p_{n}^{*}, p_{r}^{*}$)=($\frac{1-\alpha+c_{n}}{2-2\alpha}, \frac{1}{2}\beta(1-\frac{c_{n}}{(-1+\alpha)(1+(-1+\alpha)\beta^{2}\lambda+\beta(\lambda-\alpha\lambda))})$). Thus the optimal sales prices are $p_{n}^{MA*}=\frac{1-\alpha+c_{n}}{2-2\alpha}, p_{r}^{MA*}=\frac{1}{2}\beta\left( 1-\frac{c_{n}}{\left( -1+\alpha\right)\left( 1+\left( -1+\alpha\right)\beta^{2}\lambda+\beta\left( \lambda-\alpha\lambda\right) \right)} \right).$ Finally, according to the optimal resale prices, the equilibrium profits can be obtained as:$\pi_{M}^{MA*}=\frac{1}{4}\left( 1-\alpha-2c_{n}+\frac{\left( -1+\left( -1+\alpha\right)\beta\lambda\right)c_{n}^{2}}{\left( -1+\alpha\right)\left( 1+\left( -1+\alpha\right)\beta^{2}\lambda+\beta\left( \lambda-\alpha\lambda\right) \right)} \right)$,$\pi_{E}^{MA*}=\frac{1}{4}\alpha(1+\frac{\left( -1+2\left( -1+\alpha\right)\beta\lambda+\left( -1+\alpha\right)^{2}\beta^{3}\lambda^{2}-\left( -1+\alpha\right)\beta^{2}\lambda\left( 2+\left( -1+\alpha\right)\lambda\right) \right)c_{n}^{2}}{\left( -1+\alpha\right)^{2}\left( 1+\left( -1+\alpha\right)\beta^{2}\lambda+\beta\left( \lambda-\alpha\lambda\right) \right)^{2}})$.

According to the equilibrium solutions, we have:

(i). $\frac{\partial p_{n}^{MA*}}{\partial c_{n}}=\frac{1}{2-2\alpha}>0,\frac{\partial p_{r}^{MA*}}{\partial c_{n}}=-\frac{\beta}{2(-1+\alpha)(1+(-1+\alpha)\beta^{2}\lambda+\beta(\lambda-\alpha\lambda))}<0$;

(ii). $\frac{\partial p_{r}^{MA*}}{\partial\beta}=\frac{1}{2}(1+\frac{(-1+(-1+\alpha)\beta^{2}\lambda)c_{n}}{(-1+\alpha){(1+(-1+\alpha)\beta^{2}\lambda+\beta(\lambda-\alpha\lambda))}^{2}})>0$, $\frac{\partial p_{r}^{MA*}}{\partial\lambda}=\frac{\beta((1-\alpha)\beta+(-1+\alpha)\beta^{2})c_{n}}{2(-1+\alpha){(1+(-1+\alpha)\beta^{2}\lambda+\beta(\lambda-\alpha\lambda))}^{2}}<0$;

(iii).$\frac{\partial p_{n}^{MA*}}{\partial\alpha}=\frac{c_{n}}{2{(-1+\alpha)}^{2}}>0,\frac{\partial p_{r}^{MA*}}{\partial\alpha}=\frac{\beta(1-2(-1+\alpha)\beta\lambda+2(-1+\alpha)\beta^{2}\lambda)c_{n}}{2{(-1+\alpha)}^{2}{(1+(-1+\alpha)\beta^{2}\lambda+\beta(\lambda-\alpha\lambda))}^{2}}>0$. □

**Proof of Corollary 3.** In Model ER, we have the profits$\pi_{M}^{ER}$and $\pi_{E}^{ER}$.As in the proof of Proposition 1, reverse derivation is used First, we find the resale prices that maximize $\pi_{E}^{ER}$. The Hessian matrix of $\pi_{E}^{ER}$ on ($p_{n}, p_{r}$) is $H=\left[ \begin{matrix} \frac{\partial^{2}\pi_{E}^{ER}}{\partial{p_{n}}^{2}} & \frac{\partial^{2}\pi_{E}^{ER}}{\partial p_{n}p_{r}} \\ \frac{\partial^{2}\pi_{E}^{ER}}{\partial{p_{r}p}_{n}} & \frac{\partial^{2}\pi_{E}^{ER}}{\partial{p_{r}}^{2}} \end{matrix} \right]$=$\left[ \begin{matrix} \frac{-2+2(-1+\beta)\lambda}{{(-1+\beta)}^{2}\lambda} & \frac{2+2\beta\lambda-2\beta^{2}\lambda}{{(-1+\beta)}^{2}\beta\lambda} \\ \frac{2+2\beta\lambda-2\beta^{2}\lambda}{{(-1+\beta)}^{2}\beta\lambda} & \frac{2(-1-\beta\lambda+\beta^{2}\lambda)}{{(-1+\beta)}^{2}\beta^{2}\lambda} \end{matrix} \right]$, which is negatively definite. Solving the first-order conditions $\frac{\partial\pi_{E}^{MA}}{\partial p_{n}}=0$and $\frac{\partial\pi_{E}^{MA}}{\partial p_{r}}=0$for ($p_{n}^{*}, p_{r}^{*}$) = ($\frac{1}{2}\left( 1+w_{n} \right), -\frac{\beta+\beta^{2}\lambda-\beta^{3}\lambda+\beta w_{n}}{2(-1-\beta\lambda+\beta^{2}\lambda)}$). Then substitute ($p_{n}^{*}, p_{r}^{*}$) into $\pi_{M}^{ER}(w_{n})$for $w_{n}$. Differentiating $\pi_{M}^{ER}$ twice with respect to $w_{n}$, we have $\frac{{\partial^{2}\pi}_{M}^{ER}}{\partial{w_{n}}^{2}}=-\frac{2\left( 1+\beta\lambda\right)}{2+2\beta\lambda-2\beta^{2}\lambda}<0$. Solving the first-order condition $\pi_{M}^{ER}$ for $w_{n}$, we obtain the optimal wholesale price $w_{n}^{ER*}=\frac{1}{2}\left( 1+c_{n} \right)$. Bringing$w_{n}^{ER*}$ into ($p_{n}^{*}, p_{r}^{*}$) can find the optimal resale price $p_{n}^{ER*}=\frac{3+3\beta\lambda-\beta^{2}\lambda+c_{n}+\beta\lambda c_{n}}{4+4\beta\lambda}, p_{r}^{ER*}=\frac{1}{4}\beta(\frac{3+2\beta\lambda}{1+\beta\lambda}+\frac{c_{n}}{1+\beta\lambda-\beta^{2}\lambda})$. Finally, the equilibrium profits can be obtained as:$\pi_{M}^{ER*}=\frac{(-1+c_{n})(-1-\beta\lambda+\beta^{2}\lambda+(1+\beta\lambda)c_{n})}{8+8\beta\lambda-8\beta^{2}\lambda}$, $\pi_{E}^{ER*}=-\frac{1+2\beta\lambda+\beta^{2}\lambda^{2}-\beta^{4}\lambda^{2}+(-2-4\beta\lambda-2\beta^{2}(-2+\lambda)\lambda+4\beta^{3}\lambda^{2})c_{n}+{(1+\beta\lambda)}^{2}c_{n}^{2}}{16(1+\beta\lambda)(-1-\beta\lambda+\beta^{2}\lambda)}$.

According to the equilibrium solutions, we have:

(i).$\frac{\partial w_{n}^{ER*}}{\partial c_{n}}=\frac{1}{2}>0, \frac{\partial p_{n}^{ER*}}{\partial c_{n}}=\frac{1}{4}>0, \frac{\partial p_{r}^{ER*}}{\partial c_{n}}=\frac{\beta}{4+4\beta\lambda-4\beta^{2}\lambda}>0$;

(ii). $\frac{\partial p_{n}^{ER*}}{\partial\beta}=-\frac{\beta\lambda\left( 2+\beta\lambda\right)}{4\left( 1+\beta\lambda\right)^{2}}<0, \frac{\partial p_{r}^{ER*}}{\partial\beta}=\frac{1}{4}(\frac{3+4\beta\lambda+2\beta^{2}\lambda^{2}}{{(1+\beta\lambda)}^{2}}+\frac{(1+\beta^{2}\lambda)c_{n}}{{(1+\beta\lambda-\beta^{2}\lambda)}^{2}})>0$;

(iii).$\frac{\partial p_{n}^{ER*}}{\partial\lambda}=-\frac{\beta^{2}}{4\left( 1+\beta\lambda\right)^{2}}<0, \frac{\partial p_{r}^{ER*}}{\partial\lambda}=\frac{1}{4}\beta^{2}(-\frac{1}{{(1+\beta\lambda)}^{2}}+\frac{(-1+\beta)c_{n}}{{(1+\beta\lambda-\beta^{2}\lambda)}^{2}})<0.$ □

**Proof of Corollary 4.** In Model EA, we have the profits$\pi_{M}^{EA}$and $\pi_{E}^{EA}$.The Jacobi matrix of ${(\pi_{M}^{EA},\pi}_{E}^{EA})$ on ($p_{n}, p_{r}$) is $J=\left[ \begin{matrix} \frac{\partial^{2}\pi_{M}^{EA}}{\partial{p_{n}}^{2}} & \frac{\partial^{2}\pi_{M}^{EA}}{\partial p_{n}p_{r}} \\ \frac{\partial^{2}\pi_{E}^{EA}}{\partial{p_{r}p}_{n}} & \frac{\partial^{2}\pi_{E}^{EA}}{\partial{p_{r}}^{2}} \end{matrix} \right]$=$\left[ \begin{matrix} -\frac{2(-1+\alpha)}{-1+\beta} & \frac{-1+\alpha}{-1+\beta} \\ \frac{2+(1+\alpha)\beta\lambda-(1+\alpha)\beta^{2}\lambda}{{(-1+\beta)}^{2}\beta\lambda} & \frac{2(-1-\beta\lambda+\beta^{2}\lambda)}{{(-1+\beta)}^{2}\beta^{2}\lambda} \end{matrix} \right]$, which is negatively definite. Solving the first-order conditions $\frac{\partial\pi_{M}^{EA}}{\partial p_{n}}=0$and $\frac{\partial\pi_{E}^{EA}}{\partial p_{r}}=0$for ($p_{n}^{*}, p_{r}^{*}$).Thus the optimal sales prices are$p_{n}^{EA*}=\frac{2(-1-\beta\lambda+\beta^{2}\lambda)((-1+\alpha)(-1+\beta)+c_{n})}{(-1+\alpha)(4-(5+\alpha)\beta^{2}\lambda+(1+\alpha)\beta^{3}\lambda+\beta(-2+4\lambda))}, p_{r}^{EA*}=\frac{\beta(-2-(1+\alpha)\beta\lambda+(1+\alpha)\beta^{2}\lambda)((-1+\alpha)(-1+\beta)+c_{n})}{(-1+\alpha)(4-(5+\alpha)\beta^{2}\lambda+(1+\alpha)\beta^{3}\lambda+\beta(-2+4\lambda))}$. Finally, according to the optimal wholesale price and resale prices, the equilibrium profits can be obtained as: $\pi_{M}^{EA*}=\frac{(-1+\beta){(2(-1+\alpha)(-1-\beta\lambda+\beta^{2}\lambda)+(-2-2\beta\lambda+(1+\alpha)\beta^{2}\lambda)c_{n})}^{2}}{(-1+\alpha){(4-(5+\alpha)\beta^{2}\lambda+(1+\alpha)\beta^{3}\lambda+\beta(-2+4\lambda))}^{2}}$ , $\pi_{E}^{EA*}=\frac{(-1-\beta\lambda+\beta^{2}\lambda)((-1+\alpha)(-1+\beta)+c_{n})((-1+\alpha)(-((-1+\beta)\beta^{2}\lambda)+\alpha^{2}(-1+\beta)\beta^{2}\lambda+\alpha(4+4\beta\lambda-4\beta^{2}\lambda))-(\beta^{2}\lambda+\alpha^{2}\beta^{2}\lambda+2\alpha(-2-2\beta\lambda+\beta^{2}\lambda))c_{n})}{{(-1+\alpha)}^{2}(4-(5+\alpha)\beta^{2}\lambda+(1+\alpha)\beta^{3}\lambda+\beta(-2+4\lambda))}$

According to the equilibrium solutions, we have:

$\left( i \right). \frac{\partial p_{n}^{EA*}}{\partial c_{n}}=\frac{2(-1-\beta\lambda+\beta^{2}\lambda)}{(-1+\alpha)(4-(5+\alpha)\beta^{2}\lambda+(1+\alpha)\beta^{3}\lambda+\beta(-2+4\lambda))}>0$,

$\frac{\partial p_{r}^{EA*}}{\partial c_{n}}\frac{\beta(-2-(1+\alpha)\beta\lambda+(1+\alpha)\beta^{2}\lambda)}{(-1+\alpha)(4-(5+\alpha)\beta^{2}\lambda+(1+\alpha)\beta^{3}\lambda+\beta(-2+4\lambda))}>0$;

(ii). $\frac{\partial p_{n}^{EA*}}{\partial\alpha}=\frac{-((2(-1-\beta\lambda+\beta^{2}\lambda)(\left( -1+\alpha\right)^{2}\left( -1+\beta\right)^{2}\beta^{2}\lambda+2(2-(2+\alpha)\beta^{2}\lambda+\alpha\beta^{3}\lambda+\beta(-1+2\lambda))c_{n}))}{\left( -1+\alpha\right)^{2}\left( 4-\left( 5+\alpha\right)\beta^{2}\lambda+\left( 1+\alpha\right)\beta^{3}\lambda+\beta\left( -2+4\lambda\right) \right)^{2})}>0,$

$\frac{\partial p_{r}^{EA*}}{\partial\alpha}=\frac{-((\beta(4{(-1+\alpha)}^{2}{(-1+\beta)}^{2}\beta\lambda(-1-\beta\lambda+\beta^{2}\lambda)+(-8+\beta(4-16\lambda)+4\beta^{2}(5+\alpha-2\lambda)\lambda-2(5+2\alpha+\alpha^{2})\beta^{4}\lambda^{2}+{(1+\alpha)}^{2}\beta^{5}\lambda^{2}+\beta^{3}\lambda(-4+2\alpha(-2+\lambda)+17\lambda+\alpha^{2}\lambda))c_{n}))}{{(-1+\alpha)}^{2}{(4-(5+\alpha)\beta^{2}\lambda+(1+\alpha)\beta^{3}\lambda+\beta(-2+4\lambda))}^{2})}>0$;

(iii).$\frac{\partial p_{n}^{EA*}}{\partial\lambda}=\frac{2(-1+\beta)\beta^{2}((-1+\alpha)(-1+\beta)+c_{n})}{{(4-(5+\alpha)\beta^{2}\lambda+(1+\alpha)\beta^{3}\lambda+\beta(-2+4\lambda))}^{2}}<0, \frac{\partial p_{r}^{EA*}}{\partial\lambda}=\frac{4(-1+\beta)\beta^{2}((-1+\alpha)(-1+\beta)+c_{n})}{{(4-(5+\alpha)\beta^{2}\lambda+(1+\alpha)\beta^{3}\lambda+\beta(-2+4\lambda))}^{2}}<0.$ □

**Proof of Proposition 1.**$w_{n}^{MR*}-w_{r}^{MR*}=\frac{1}{2}\left( 1+c_{n}+\frac{\beta\left( 1+2\beta\lambda-2\beta^{2}\lambda+c_{n} \right)}{-1-2\beta\lambda+2\beta^{2}\lambda} \right)=\Delta$ , differentiating $\Delta$ with respect to $\lambda$, we have $\frac{\partial\Delta}{\partial\lambda}=-\frac{\left( -1+\beta\right)\beta^{2}c_{n}}{\left( 1+2\beta\lambda-2\beta^{2}\lambda\right)^{2}}>0$, i.e., $\Delta$ is increased in $\lambda$. Letting $\lambda=0,$we have $\Delta= -\frac{1}{2}\left( -1+\beta\right)\left( 1+c_{n} \right)>0$, i.e.,$\Delta$> 0. Thus $w_{n}^{MR*}>w_{r}^{MR*}$. □

**Proof of Proposition 2.** (i).$p_{n}^{MR*}-p_{n}^{MA*}=\frac{-1+\alpha+\left( 1+\alpha\right)c_{n}}{4\left( -1+\alpha\right)}=\Delta$, differentiating $\Delta$ with respect to $c_{n}$, we have $\frac{\partial\Delta}{\partial c_{n}}=\frac{1+\alpha}{4(-1+\alpha)}<0$, i.e., $\Delta$ is decreased in $c_{n}$. Solving $\Delta=0,$we get $c_{n}=\frac{1-\alpha}{1+\alpha}$=$c_{n1}>0$. So $p_{n}^{MR*}>p_{n}^{MA*}, when c_{n}<c_{n1}$.

(ii). $p_{n}^{ER*}-p_{n}^{EA*}=-\frac{2\left( -1-\beta\lambda+\beta^{2}\lambda\right)\left( \left( -1+\alpha\right)\left( -1+\beta\right)+c_{n} \right)}{\left( -1+\alpha\right)\left( 4-\left( 5+\alpha\right)\beta^{2}\lambda+\left( 1+\alpha\right)\beta^{3}\lambda+\beta\left( -2+4\lambda\right) \right)}+\frac{3+3\beta\lambda-\beta^{2}\lambda+c_{n}+\beta\lambda c_{n}}{4+4\beta\lambda}=\Delta,$ differentiating $\Delta$ with respect to $c_{n}$, we have $\frac{\partial\Delta}{\partial c_{n}}=\frac{1}{4}+\frac{2+2\beta\lambda-2\beta^{2}\lambda}{(-1+\alpha)(4-(5+\alpha)\beta^{2}\lambda+(1+\alpha)\beta^{3}\lambda+\beta(-2+4\lambda))}<0$, i.e., $\Delta$ is decreased in $c_{n}$. Solving $\Delta=0,$we get $c_{n}=\frac{(-1+\alpha)(-4+\beta^{2}(1+3\alpha-4\lambda)\lambda-4\alpha\beta^{4}\lambda^{2}+(1+\alpha)\beta^{5}\lambda^{2}+3\beta^{3}\lambda(1+\alpha(-1+\lambda)+\lambda)-2\beta(1+4\lambda))}{(1+\beta\lambda)(4-3\beta^{2}\lambda+\alpha^{2}(-1+\beta)\beta^{2}\lambda-\beta^{3}\lambda+\beta(2+4\lambda)-2\alpha(-2+\beta-2\beta\lambda+2\beta^{2}\lambda))}$=$c_{n2}>0$. So $p_{n}^{ER*}>p_{n}^{EA*}, when c_{n}<c_{n2}$.

(ii).$p_{n}^{MR*}-p_{n}^{ER*}=\frac{\beta^{2}\lambda}{4+4\beta\lambda}>0$,i.e, $p_{n}^{MR*}>p_{n}^{ER*}$;$p_{n}^{MA*}-p_{n}^{EA*}=\frac{1-\alpha+c_{n}}{2-2\alpha}-\frac{2(-1-\beta\lambda+\beta^{2}\lambda)((-1+\alpha)(-1+\beta)+c_{n})}{(-1+\alpha)(4-(5+\alpha)\beta^{2}\lambda+(1+\alpha)\beta^{3}\lambda+\beta(-2+4\lambda))}=\Delta,$ differentiating $\Delta$ with respect to $c_{n}$, we have $\frac{\partial\Delta}{\partial c_{n}}=\frac{\beta(2+(1+\alpha)\beta\lambda-(1+\alpha)\beta^{2}\lambda)}{2(-1+\alpha)(4-(5+\alpha)\beta^{2}\lambda+(1+\alpha)\beta^{3}\lambda+\beta(-2+4\lambda))}<0$, i.e., $\Delta$ is decreased in $c_{n}$. Solving $\Delta=0,$we get $c_{n}=\frac{(-1+\alpha)(2-(-3+\alpha)\beta\lambda+(-3+\alpha)\beta^{2}\lambda)}{-2-(1+\alpha)\beta\lambda+(1+\alpha)\beta^{2}\lambda}$=$c_{n3}>0$. So $p_{n}^{MA*}>p_{n}^{EA*}, when c_{n}<c_{n3}$.

(iii). $p_{r}^{MR*}-p_{r}^{MA*}=\frac{1}{2}\beta(\frac{1}{2}+(\frac{1}{2+4\beta\lambda-4\beta^{2}\lambda}+\frac{1}{(-1+\alpha)(1+(-1+\alpha)\beta^{2}\lambda+\beta(\lambda-\alpha\lambda))})c_{n})=\Delta,$ differentiating $\Delta$ with respect to $c_{n}$, we have $\frac{\partial\Delta}{\partial c_{n}}=\frac{1}{2}\beta(\frac{1}{2+4\beta\lambda-4\beta^{2}\lambda}+\frac{1}{(-1+\alpha)(1+(-1+\alpha)\beta^{2}\lambda+\beta(\lambda-\alpha\lambda))})<0$, i.e., $\Delta$ is decreased in $c_{n}$. Solving $\Delta=0,$we get $c_{n}=\frac{1-\alpha+3\beta\lambda-4\alpha\beta\lambda+\alpha^{2}\beta\lambda-3\beta^{2}\lambda+4\alpha\beta^{2}\lambda-\alpha^{2}\beta^{2}\lambda+2\beta^{2}\lambda^{2}-4\alpha\beta^{2}\lambda^{2}+2\alpha^{2}\beta^{2}\lambda^{2}-4\beta^{3}\lambda^{2}+8\alpha\beta^{3}\lambda^{2}-4\alpha^{2}\beta^{3}\lambda^{2}+2\beta^{4}\lambda^{2}-4\alpha\beta^{4}\lambda^{2}+2\alpha^{2}\beta^{4}\lambda^{2}}{1+\alpha+3\beta\lambda+2\alpha\beta\lambda-\alpha^{2}\beta\lambda-3\beta^{2}\lambda-2\alpha\beta^{2}\lambda+\alpha^{2}\beta^{2}\lambda}$=$c_{n4}>0$. So $p_{r}^{MR*}>p_{r}^{MA*}, when c_{n}<c_{n4}$.$p_{r}^{ER*}-p_{r}^{EA*}=\frac{1}{4}\beta(\frac{3+2\beta\lambda}{1+\beta\lambda}+\frac{c_{n}}{1+\beta\lambda-\beta^{2}\lambda}-\frac{4(-2-(1+\alpha)\beta\lambda+(1+\alpha)\beta^{2}\lambda)((-1+\alpha)(-1+\beta)+c_{n})}{(-1+\alpha)(4-(5+\alpha)\beta^{2}\lambda+(1+\alpha)\beta^{3}\lambda+\beta(-2+4\lambda))}=\Delta,$ differentiating $\Delta$ with respect to $c_{n}$, we have $\frac{\partial\Delta}{\partial c_{n}}=\frac{1}{4}\beta(\frac{1}{1+\beta\lambda-\beta^{2}\lambda}+\frac{8+4(1+\alpha)\beta\lambda-4(1+\alpha)\beta^{2}\lambda}{(-1+\alpha)(4-(5+\alpha)\beta^{2}\lambda+(1+\alpha)\beta^{3}\lambda+\beta(-2+4\lambda))})<0$, i.e., $\Delta$ is decreased in $c_{n}$. Solving $\Delta=0,$we get $c_{n}=\frac{4(-\frac{\beta(3+2\beta\lambda)}{4+4\beta\lambda}+\frac{(-1+\beta)\beta(-2-(1+\alpha)\beta\lambda+(1+\alpha)\beta^{2}\lambda)}{4-(5+\alpha)\beta^{2}\lambda+(1+\alpha)\beta^{3}\lambda+\beta(-2+4\lambda)})}{\beta(\frac{1}{1+\beta\lambda-\beta^{2}\lambda}+\frac{8+4(1+\alpha)\beta\lambda-4(1+\alpha)\beta^{2}\lambda}{(-1+\alpha)(4-(5+\alpha)\beta^{2}\lambda+(1+\alpha)\beta^{3}\lambda+\beta(-2+4\lambda))})}$=$c_{n5}>0$. So $p_{r}^{ER*}>p_{r}^{EA*}, when c_{n}<c_{n5}$.

(iv).$p_{r}^{MR*}-p_{r}^{ER*}=\beta(\frac{\beta\lambda}{4+4\beta\lambda}+(\frac{1}{4+8\beta\lambda-8\beta^{2}\lambda}+\frac{1}{-4-4\beta\lambda+4\beta^{2}\lambda})c_{n})=\Delta,$ differentiating $\Delta$ with respect to $c_{n}$, we have $\frac{\partial\Delta}{\partial c_{n}}=\beta(\frac{1}{4+8\beta\lambda-8\beta^{2}\lambda}+\frac{1}{-4-4\beta\lambda+4\beta^{2}\lambda})<0$, i.e., $\Delta$ is decreased in $c_{n}$. Solving $\Delta=0,$we get $c_{n}=-\frac{(-1-\beta\lambda+\beta^{2}\lambda)(-1-2\beta\lambda+2\beta^{2}\lambda)}{(-1+\beta)(1+\beta\lambda)}$=$c_{n6}>0$. So $p_{r}^{MR*}>p_{r}^{ER*}, when c_{n}<c_{n6}$.$p_{r}^{MA*}-p_{r}^{EA*}=\frac{1}{2}\beta(1-\frac{c_{n}}{(-1+\alpha)(1+(-1+\alpha)\beta^{2}\lambda+\beta(\lambda-\alpha\lambda))}-\frac{2(-2-(1+\alpha)\beta\lambda+(1+\alpha)\beta^{2}\lambda)((-1+\alpha)(-1+\beta)+c_{n})}{(-1+\alpha)(4-(5+\alpha)\beta^{2}\lambda+(1+\alpha)\beta^{3}\lambda+\beta(-2+4\lambda))})=\Delta,$ differentiating $\Delta$ with respect to $c_{n}$, we have $\frac{\partial\Delta}{\partial c_{n}}=\frac{\beta(\frac{4+2(1+\alpha)\beta\lambda-2(1+\alpha)\beta^{2}\lambda}{4-(5+\alpha)\beta^{2}\lambda+(1+\alpha)\beta^{3}\lambda+\beta(-2+4\lambda)}-\frac{1}{1+(-1+\alpha)\beta^{2}\lambda+\beta(\lambda-\alpha\lambda)})}{2(-1+\alpha)}<0$, i.e., $\Delta$ is decreased in $c_{n}$. Solving $\Delta=0,$we get $c_{n}=-\frac{((-1+\alpha)(-2+(-1+\beta)(2+\alpha(-2+\beta)+\beta)\lambda)(1+(-1+\alpha)\beta^{2}\lambda+\beta(\lambda-\alpha\lambda))}{-2+(-1+\beta)(2+\alpha(-2+\beta)+\beta)\lambda+2(-1+\alpha^{2}){(-1+\beta)}^{2}\beta\lambda^{2}}$=$c_{n7}>0$. So $p_{r}^{MA*}>p_{r}^{EA*}, when c_{n}<c_{n7}$. □

**Proof of Proposition 3.** (i).$\pi_{M}^{MR*}-\pi_{M}^{MA*}=\frac{1}{8}\left( \frac{-1-2\beta\lambda+2\beta^{2}\lambda+\left( 2+4\beta\lambda-4\beta^{2}\lambda\right)c_{n}-\left( 1+2\beta\lambda\right)c_{n}^{2}}{-1-2\beta\lambda+2\beta^{2}\lambda}+2\left( -1+\alpha+2c_{n}-\frac{\left( -1+\left( -1+\alpha\right)\beta\lambda\right)c_{n}^{2}}{\left( -1+\alpha\right)\left( 1+\left( -1+\alpha\right)\beta^{2}\lambda+\beta\left( \lambda-\alpha\lambda\right) \right)} \right) \right)=\Delta,$ differentiating$\Delta$ with respect to $\lambda$, we have$\frac{\partial\Delta}{\partial\lambda}=\frac{(1+\alpha)(-1+\beta)\beta^{3}\lambda(2-(-3+\alpha)\beta\lambda+(-3+\alpha)\beta^{2}\lambda)c_{n}^{2}}{4{(1+2\beta\lambda-2\beta^{2}\lambda)}^{2}{(1+(-1+\alpha)\beta^{2}\lambda+\beta(\lambda-\alpha\lambda))}^{2}}<0$,i.e., $\pi_{M}^{MR*}-\pi_{M}^{MA*}$ is decreased in $\lambda$.So $\pi_{M}^{MR*}>\pi_{M}^{MA*}$ when $\lambda$is low, $\pi_{M}^{MR*}<\pi_{M}^{MA*}$ when $\lambda$is high.

(ii). $\pi_{M}^{ER*}-\pi_{M}^{EA*}=\frac{(-1+c_{n})(-1-\beta\lambda+\beta^{2}\lambda+(1+\beta\lambda)c_{n})}{8+8\beta\lambda-8\beta^{2}\lambda}-\frac{(-1+\beta){(2(-1+\alpha)(-1-\beta\lambda+\beta^{2}\lambda)+(-2-2\beta\lambda+(1+\alpha)\beta^{2}\lambda)c_{n})}^{2}}{(-1+\alpha){(4-(5+\alpha)\beta^{2}\lambda+(1+\alpha)\beta^{3}\lambda+\beta(-2+4\lambda))}^{2}}=\Delta,$ differentiating$\Delta$ with respect to $\alpha$, we have$\frac{\partial\Delta}{\partial\alpha}=A$. Differentiating$A$ with respect to $c_{n}$, we have$\frac{\partial A}{\partial c_{n}}=C<0,$i.e., $A$ is decreasing in $c_{n}$. Solving $A=0,$ we get $c_{n}=-\frac{2(-1+\alpha)(-1-\beta\lambda+\beta^{2}\lambda)}{-2-2\beta\lambda+(1+\alpha)\beta^{2}\lambda}$=$c_{n8}>0$. Letting $c_{n}=0$we have $A=\frac{4(-1+\beta){(-1-\beta\lambda+\beta^{2}\lambda)}^{2}(-4+\beta(2-4\lambda)-(-7+\alpha)\beta^{2}\lambda+(-3+\alpha)\beta^{3}\lambda)}{{(4-(5+\alpha)\beta^{2}\lambda+(1+\alpha)\beta^{3}\lambda+\beta(-2+4\lambda))}^{3}}>0$. So we can get that $\frac{\partial\Delta}{\partial\alpha}=A>0$when $c_{n}<c_{n8}$,$\frac{\partial\Delta}{\partial\alpha}=A<0$when $c_{n}>c_{n8}$, which means ${\Delta=\pi}_{M}^{ER*}-\pi_{M}^{EA*}$is positively related to $\alpha$when $c_{n}<c_{n8}$, else $\Delta$is negatively related to $\alpha$. To summarize, it can be concluded that ①.$c_{n}<c_{n8}, \pi_{M}^{ER*}>\pi_{M}^{EA*}$ when $\alpha$ is high, $\pi_{M}^{ER*}<\pi_{M}^{EA*}$ when $\alpha$ is low; ②. $c_{n}>c_{n8}, \pi_{M}^{ER*}>\pi_{M}^{EA*}$ when $\alpha$ is low, $\pi_{M}^{ER*}<\pi_{M}^{EA*}$ when $\alpha$ is high. □

**Proof of Proposition 4.** $\pi_{M}^{MR*}-\pi_{M}^{ER*}=\frac{\beta^{2}\lambda c_{n}(1+2\beta\lambda-2\beta^{2}\lambda+c_{n})}{8(-1-\beta\lambda+\beta^{2}\lambda)(-1-2\beta\lambda+2\beta^{2}\lambda)}$=$\Delta,$differentiating $\Delta$ with respect to $c_{n}$, we have $\frac{\partial\Delta}{\partial c_{n}}=\frac{\beta^{2}\lambda(1+2\beta\lambda-2\beta^{2}\lambda+2c_{n})}{8(-1-\beta\lambda+\beta^{2}\lambda)(-1-2\beta\lambda+2\beta^{2}\lambda)}>0$, i.e., $\Delta$ is increasing in $c_{n}$. Letting $c_{n}=0,$we have $\Delta= 0$. Cause $c_{n}>c_{r}=0,\pi_{M}^{MR*}>\pi_{M}^{ER*}$.

$\pi_{M}^{MA*}-\pi_{M}^{EA*}=\frac{1}{4}(1-\alpha-2c_{n}+\frac{\left( -1+\left( -1+\alpha\right)\beta\lambda\right)c_{n}^{2}}{\left( -1+\alpha\right)\left( 1+\left( -1+\alpha\right)\beta^{2}\lambda+\beta\left( \lambda-\alpha\lambda\right) \right)}-\frac{4(-1+\beta){(2(-1+\alpha)(-1-\beta\lambda+\beta^{2}\lambda)+(-2-2\beta\lambda+(1+\alpha)\beta^{2}\lambda)c_{n})}^{2}}{(-1+\alpha){(4-(5+\alpha)\beta^{2}\lambda+(1+\alpha)\beta^{3}\lambda+\beta(-2+4\lambda))}^{2}})$ =$\Delta,$differentiating $\Delta$ with respect to $c_{n}$, we have $\frac{\partial\Delta}{\partial c_{n}}=C$,$C>0$when $c_{n}>\hat{c_{n}}$. Letting $c_{n}=\hat{c_{n}}$, we have $\Delta>0$. Letting $c_{n}=0$, we have $\Delta>0$. So $\pi_{M}^{MA*}>\pi_{M}^{EA*}$. □

**Proof of Proposition 5.**(i). $\pi_{E}^{MR*}-\pi_{E}^{MA*}=\frac{1}{16}(1-2c_{n}+\frac{\left( 1+4\beta\lambda+4\beta^{2}\left( -1+\lambda\right)\lambda-4\beta^{3}\lambda^{2} \right)c_{n}^{2}}{\left( 1+2\beta\lambda-2\beta^{2}\lambda\right)^{2}}-4\alpha(1+\frac{(-1+2(-1+\alpha)\beta\lambda+{(-1+\alpha)}^{2}\beta^{3}\lambda^{2}-(-1+\alpha)\beta^{2}\lambda(2+(-1+\alpha)\lambda))c_{n}^{2}}{{(-1+\alpha)}^{2}{(1+(-1+\alpha)\beta^{2}\lambda+\beta(\lambda-\alpha\lambda))}^{2}}))=\Delta,$ differentiating$\Delta$ with respect to $\lambda$, we have$\frac{\partial\Delta}{\partial\lambda}=\frac{(-1+\beta)\beta^{3}\lambda(\alpha^{3}{(-1+\beta)}^{3}\beta^{3}\lambda^{3}-3\alpha^{2}{(-1+\beta)}^{2}\beta^{2}\lambda^{2}(-1-\beta\lambda+\beta^{2}\lambda)-{(-1-\beta\lambda+\beta^{2}\lambda)}^{3}+\alpha(1+3\beta\lambda+3\beta^{4}(2-5\lambda)\lambda^{2}+15\beta^{5}\lambda^{3}-5\beta^{6}\lambda^{3}+3\beta^{2}\lambda(-1+2\lambda)+\beta^{3}\lambda^{2}(-12+5\lambda)))c_{n}^{2}}{2{(-1-2\beta\lambda+2\beta^{2}\lambda)}^{3}{(1+(-1+\alpha)\beta^{2}\lambda+\beta(\lambda-\alpha\lambda))}^{3}}>0$,i.e., $\pi_{M}^{MR*}-\pi_{M}^{MA*}$ is increased in $\lambda$.So $\pi_{E}^{MR*}>\pi_{E}^{MA*}$ when $\lambda$is high, $\pi_{M}^{MR*}<\pi_{M}^{MA*}$ when $\lambda$is low.

(ii). $\pi_{E}^{ER*}-\pi_{E}^{EA*}=-\frac{(-1-\beta\lambda+\beta^{2}\lambda)((-1+\alpha)(-1+\beta)+c_{n})((-1+\alpha)(-((-1+\beta)\beta^{2}\lambda)+\alpha^{2}(-1+\beta)\beta^{2}\lambda+\alpha(4+4\beta\lambda-4\beta^{2}\lambda))-(\beta^{2}\lambda+\alpha^{2}\beta^{2}\lambda+2\alpha(-2-2\beta\lambda+\beta^{2}\lambda))c_{n})}{{(-1+\alpha)}^{2}{(4-(5+\alpha)\beta^{2}\lambda+(1+\alpha)\beta^{3}\lambda+\beta(-2+4\lambda))}^{2}}-\frac{1+2\beta\lambda+\beta^{2}\lambda^{2}-\beta^{4}\lambda^{2}+(-2-4\beta\lambda-2\beta^{2}(-2+\lambda)\lambda+4\beta^{3}\lambda^{2})c_{n}+{(1+\beta\lambda)}^{2}c_{n}^{2}}{16(1+\beta\lambda)(-1-\beta\lambda+\beta^{2}\lambda)}=\Delta,$ differentiating$\Delta$ with respect to $\alpha$, we have$\frac{\partial\Delta}{\partial\alpha}=A$. Solving $A=0,$we have $c_{n}=c_{n10}>0$. Letting $c_{n}=0$, we have $A<0$.So $\frac{\partial\Delta}{\partial\alpha}=A<0$when$c_{n}<c_{n10}$and $\frac{\partial\Delta}{\partial\alpha}=A>0$when$c_{n}>c_{n9}$.So $\pi_{E}^{ER*}-\pi_{E}^{EA*}$is decreasing in $\alpha$when $c_{n}<c_{n9}$, else $\pi_{E}^{ER*}-\pi_{E}^{EA*}$is increasing in $\alpha$when $c_{n}<c_{n9}$ .Thus we have ①.$c_{n}<c_{n9}, \pi_{E}^{ER*}>\pi_{E}^{EA*}$ when $\alpha$is low, $\pi_{E}^{ER*}<\pi_{E}^{EA*}$ when $\alpha$is high; ②.$c_{n}>c_{n9}, \pi_{E}^{ER*}>\pi_{E}^{EA*}$ when $\alpha$is high, $\pi_{E}^{ER*}<\pi_{E}^{EA*}$ when $\alpha$is low.□

**Proof of Proposition 6.** $\pi_{M}^{MR*}-\pi_{M}^{ER*}=\frac{1}{16}\beta^{2}\lambda\left( -\frac{1}{1+\beta\lambda}+\frac{2c_{n}}{-1-\beta\lambda+\beta^{2}\lambda}+\frac{c_{n}^{2}}{\left( 1+2\beta\lambda-2\beta^{2}\lambda\right)^{2}\left( -1-\beta\lambda+\beta^{2}\lambda\right)} \right)$. According to the range of values of the parameter:$0<\beta<1, 0<\lambda<1, c_{n}>0,$it can be concluded that$\pi_{M}^{MR*}-\pi_{M}^{ER*}>0,$i.e.,$\pi_{M}^{MR*}<\pi_{M}^{ER*}$.
